# Integrative approach to sporadic Alzheimer’s disease: deficiency of TYROBP in cerebral Aβ amyloidosis mouse normalizes clinical phenotype and complement subnetwork molecular pathology without reducing Aβ burden

**DOI:** 10.1038/s41380-018-0255-6

**Published:** 2018-10-03

**Authors:** Jean-Vianney Haure-Mirande, Minghui Wang, Mickael Audrain, Tomas Fanutza, Soong Ho Kim, Szilvia Heja, Ben Readhead, Joel T. Dudley, Robert D. Blitzer, Eric E. Schadt, Bin Zhang, Sam Gandy, Michelle E. Ehrlich

**Affiliations:** 1 Department of Neurology, Icahn School of Medicine at Mount Sinai, New York, NY 10029 USA; 2 Department of Genetics and Genomic Sciences and Icahn Institute of Genomic Sciences, Icahn School of Medicine at Mount Sinai, New York, NY 10029 USA; 3 Departments of Pharmacological Sciences and Psychiatry, Icahn School of Medicine at Mount Sinai, New York, NY 10029 USA; 4 Sema4, a Mount Sinai venture, Stamford, CT 06902 USA; 5 Department of Psychiatry and Alzheimer’s Disease Research Center, Icahn School of Medicine at Mount Sinai, New York, NY 10029 USA; 6 Department of Pediatrics, Icahn School of Medicine at Mount Sinai, New York, NY 10029 USA

**Keywords:** Molecular biology, Neuroscience

## Abstract

Integrative gene network approaches enable new avenues of exploration that implicate causal genes in sporadic late-onset Alzheimer’s disease (LOAD) pathogenesis, thereby offering novel insights for drug-discovery programs. We previously constructed a probabilistic causal network model of sporadic LOAD and identified *TYROBP/DAP12*, encoding a microglial transmembrane signaling polypeptide and direct adapter of TREM2, as the most robust key driver gene in the network. Here, we show that absence of TYROBP/DAP12 in a mouse model of AD-type cerebral Aβ amyloidosis (*APP*^*KM670/671NL*^/*PSEN1*^*Δexon9*^) recapitulates the expected network characteristics by normalizing the transcriptome of *APP/PSEN1* mice and repressing the induction of genes involved in the switch from homeostatic microglia to disease-associated microglia (DAM), including *Trem2*, complement (*C1qa*, *C1qb*, *C1qc*, and *Itgax*), *Clec7a* and *Cst7*. Importantly, we show that constitutive absence of TYROBP/DAP12 in the amyloidosis mouse model prevented appearance of the electrophysiological and learning behavior alterations associated with the phenotype of *APP*^*KM670/671NL*^/*PSEN1*^*Δexon9*^ mice. Our results suggest that *TYROBP/DAP12* could represent a novel therapeutic target to slow, arrest, or prevent the development of sporadic LOAD. These data establish that the network pathology observed in postmortem human LOAD brain can be faithfully recapitulated in the brain of a genetically manipulated mouse. These data also validate our multiscale gene networks by demonstrating how the networks intersect with the standard neuropathological features of LOAD.

## Introduction

Genome-wide association studies (GWAS) have identified two dozen genes that can be associated with causation or increased risk for sporadic late-onset Alzheimer’s disease (hereafter referred as LOAD) [1–3], the major cause of late-life brain failure in humans [4]. Large GWAS datasets [1–3] point to molecules in one of three general classes: (1) microglial or immune-inflammatory genes; (2) protein processing and/or sorting genes; or (3) lipid- or lipoprotein-related genes. Such datasets indicate many of the molecules that can be prominently perturbed in LOAD [5–16]. Bioinformatic analysis of genome-wide RNA expression provides insights into biological pathways that regulate cellular processes and disease progression at the molecular level and is widely applied to investigate the pathogenesis of familial AD (FAD) and LOAD in mouse models and in human postmortem brain [5–17]. Gene co-expression network analysis approaches capture interactions among genes and identify higher order network structures, e.g., modules comprising highly interconnected genes [5–16]. Zhang et al. [5] performed a multiscale gene network analysis (MNA) combining neuropathology, whole-genome genotyping, and gene-expression profiling of human brain specimens from visual cortex, dorsolateral prefrontal cortex (PFC), and cerebellum from 376 AD patients and 173 nondemented controls. MNA revealed many facets of the molecular-interaction structures in AD and formally rank-ordered gene subnetworks based on their relevance to AD pathological and clinical traits. One such subnetwork highlighted complement cascade-related molecules, and the key complement subnetwork driver was *TYROBP* (also known as *DAP12*), a phagocytosis-related, microglial-enriched, phosphotyrosine phosphoprotein that forms functional complexes with, among others, TREM2 and the complement receptor CR3 [18–22]. In addition to its identity as a subnetwork driver, loss-of-function mutant forms of *TYROBP* lead to Nasu-Hakola disease [23], while rare missense forms may result in FAD [24]. Beyond the association of TYROBP with LOAD and FAD, TREM2, and CR3 have well-documented interactions with amyloid peptide (Aβ), complement, and synapses [25–27]. Notably, there exists an important phenomenon whereby oligomeric forms of Aβ trigger the engulfment of synaptic structures via a CR3-dependent process [27]. Perhaps because both CR3 and TYROBP are obligatory participants in this phenomenon, mice deficient in either C3 (the ligand for CR3) or TYROBP are relatively resistant to Aβ-induced behavioral and electrophysiological pathology even at the young age of 4 months [26, 28].

We sought to validate in vivo in mice the driver role of *TYROBP* in LOAD and to demonstrate that the manipulation of *Tyrobp* leads to changes in subnetworks that mimic the changes observed in the brain in human LOAD. We previously demonstrated that 4-month-old transgenic *APP*^*KM670/671NL*^/*PSEN1*^*Δexon9*^ (hereafter abbreviated as *APP/PSEN1*) mice on a *Tyrobp*-null (*Tyrobp*^−/−^) background had no apparent changes in the transcription of the constituents of the complement subnetwork [28]. Since all forms of AD are  aging-related, we performed the identical battery of molecular, behavioral, electrophysiological, and transcriptomic analyses in 8-month-old mice. As observed in 4-month-old mice, TYROBP deficiency played a protective effect in behavior and electrophysiology in 8-month-old mice. However, unlike the 4-month-old mice, we observed that the levels of the transcripts comprising nearly the entire *Tyrobp*-driven complement subnetwork were reduced. Therefore, constitutive *Tyrobp* knockout in the brain of a mouse with Aβ-amyloid deposits, recapitulates, in an aging-related manner, the complement subnetwork first observed in human LOAD brain [5]. As one would expect, the transcriptomic subnetwork and hub that were associated with *increased* expression in LOAD brain were associated with *reduced* representation in the *APP/PSEN1*;*Tyrobp*^−/−^ brain.

This is the first direct observation that the complement transcriptome subnetwork molecules predicted from integrative models of human postmortem LOAD brain can be recapitulated in a cerebral amyloidosis mouse model. Moreover, the levels of network component transcripts are reversed in a mouse in which the predicted driver has been knocked out. The ability of a single subnetwork hub/driver to prevent the appearance of the typical behavioral and electrophysiological phenotypes of the *APP/PSEN1* mouse while recapitulating the predicted subnetwork characteristics can now serve as a biological template for designing, screening, and repurposing molecular interventions in an effort to identify a biologic or a small molecule drug capable of mimicking the profile of the 8-month-old, Aβ-amyloid-depositing, *Tyrobp* knockout mouse. Our prediction is that drugs identified in this manner may be useful in the prevention or treatment of LOAD.

## Methods

### Animals

The experimental procedures were conducted in accordance with NIH guidelines for animal research and were approved by the Institutional Animal Care and Use Committee (IACUC) at Icahn School of Medicine at Mount Sinai. All mice were on a C57Bl6/J background. *APP*^*KM670/671NL*^/*PSEN1*^*Δexon9*^ (=*APP/PSEN1*) [29], *Tyrobp-*null or knockout (*Tyrobp*^−/−^) [30] were obtained from Jackson Laboratories and Taconic/Merck Laboratory respectively. *APP/PSEN1* were crossed with *Tyrobp*^−/−^ mice to obtain *APP/PSEN1;Tyrobp*^*+/−*^. *APP/PSEN1;Tyrobp*^*+/*−^ were then crossed with *Tyrobp*^*+/*−^ to obtain WT, *Tyrobp*^*+/−*^, *Tyrobp*^−/−^, *APP/PSEN1*, *APP/PSEN1;Tyrobp*^*+/*−^ or *APP/PSEN1;Tyrobp*^−/−^ mice. The 8-month-old male and female mice were sacrificed by decapitation. One hemisphere was collected and immersion-fixed in 4% paraformaldehyde for immunohistochemistry analysis. The other hemisphere was dissected and prefrontal cortex (PFC)  was collected for transcriptomic analysis. PFC and hemibrain were then snap-frozen and stored at −80 °C prior to RNA isolation or biochemistry analysis. A standard number of mice (4–5 per group and 4–5 per sex) were used for most of the analyses as this size gives us 80% power to see differences of effect size of at least 2.5 between groups at *α* = 0.05. An increased standard number of mice were used per group and per sex for behavioral and electrophysiological analyses as these analyses are often variable (sample size “*n*” is specified in the figure legend).

### RNA isolation

Snap frozen samples from the PFC of male and female mice were homogenized in QIAzol Lysis Reagent (Qiagen). Total RNA purification was performed with the miRNeasy Mini kit (Qiagen). RNA quantification and quality were evaluated by Agilent BioAnalyzer. RNA integrity was checked using the RNA 6000 Nano assay (Agilent, CA, USA). All processed RNA samples had RQN/RIN value of 8.4 or greater.

### RT-qPCR

500 ng of total RNAs were reversed transcribed using the High-Capacity RNA-to-cDNA Kit (Applied Biosystem, Foster City, CA, USA). cDNAs were subjected to real-time qPCR in a Step­One Plus system (Applied Biosystem) using The All-in-One qPCR Mix (GeneCopoeia, Rockville, MD, USA). qPCR consisted of 40 cycles, 10 s at 95 °C, 20 s at 60 °C, and 15 s at 70 °C each, followed by dissociation curve analysis. Ct values were normalized to the expression level of L32 and relative gene expression was calculated using the ΔΔCt method [31]. Sequences of primers used:

C1q: Fwd5′-GCATCCAGTTTGATCGGACC-3’; Rev5’-GCTCCCCTCTCTCTCCTTTG-3′

L32: Fwd5′-GAAACTGGCGGAAACCCA-3′; Rev5′-GGATCTGGCCCTTGAACCTT-3′

### RNA sequencing

Total RNAs from PFC of 8-month-old male WT (*n* = 4), *Tyrobp*^*+/*^^−^ (*n* = 3), *Tyrobp*^−/−^(*n* = 4), *APP/PSEN1* (*n* = 4), *APP/PSEN1;Tyrobp*^*+/−*^ (*n* = 4) or *APP/PSEN1;Tyrobp*^−/−^ (*n* = 5) mice were subjected to RNA sequencing. The sequencing library was prepared with the TruSeq RNA Sample Prep Kit v2 protocol (Illumina, CA, USA). Ribosomal RNAs were removed using the Ribo-Zero rRNA Removal Kit (Human/Mouse/Rat) (Illumina, CA, USA). Remaining RNAs were fragmented, and the cDNAs synthesized using random hexamers, end-repaired, and ligated with appropriate adapters for sequencing. The library was processed for size selection and purification using AMPure XP beads (Beckman Coulter, CA, USA). The appropriate Illumina-recommended 6 bp barcode bases were introduced at one end of the adapters during the PCR amplification step. The size and concentration of the RNA-seq libraries were measured by Bioanalyzer and Qubit fluorometry (Life Technologies, NY, USA) before loading onto the sequencer. The rRNA-depleted libraries were sequenced on the Illumina HiSeq 2500 System with 100 nucleotide single-end reads (Illumina, CA, USA).

### Differential expression analysis and gene set enrichment analysis

The single-ended raw sequencing reads were aligned to mouse mm10 genome using Star aligner (version 2.5.0b). FeatureCounts was used to quantify gene expression at the gene level based on UCSC gene model. Genes with at least 1 count per million in at least one sample were considered expressed and retained for further analysis. The gene level read count data were normalized using trimmed mean of the *M*-values normalization method [32] to adjust for sequencing library size difference. Differential gene expression between groups was predicted by a linear model analysis using Bioconductor package limma [33]. To adjust for multiple tests, the false discovery rate (FDR) of the differential expression test was estimated using the Benjamini–Hochberg method [34]. Gene set enrichments were calculated using ingenuity pathway analysis (IPA).

### Network analysis using human AD postmortem brain gene-expression data

We utilized large-scale postmortem brain RNA sequencing data from two human AD cohorts (MSBB [6, 35] and ROSMAP [36]) to build gene co-expression networks and Bayesian causal networks to model the regulation of gene-expression traits in brain samples. The ROSMAP and MSBB RNA-seq data and associated genomics data are available in the Accelerating Medicines Partnership in AD (AMP-AD) Knowledge Portal at Synapse (https://www.synapse.org/#!Synapse:syn2580853) upon authentication by the AMP-AD Consortium. We downloaded normalized RNA-seq gene-expression abundance data from Synapse. Genes expressed in at least 10% of the samples were selected, and the data were corrected for confounding factors including batch, PMI, sex, and RIN score. The construction of gene co-expression networks and Bayesian networks is detailed in Suppl. methods.

### Immunohistochemistry

30µm thick  sagittal free-floating sections were incubated with anti-Iba1 (1:500, cat#019-19741, Wako, Richmond, VA, USA), 6E10 (1:1000, cat#9320-500, Covance, Princeton, NJ, USA), or C1q (1:1000, cat#ab182451, Abcam, Cambridge, MA, USA) antibodies. Sections probed with Iba1 and 6E10 antibodies were incubated with anti-rabbit Alexa 488 (1:400, cat#A-11008, Thermo Fisher Scientific, Grand Island, NY, USA) and anti-mouse Alexa 594 (1:400, cat#A-11005, Thermo Fisher Scientific) secondary antibodies. Sections probed with C1q were incubated with biotinylated anti-mouse antibody (1:1000, BA-9200, Vector laboratories, Burlingame, CA, USA) and developed with Vectastain ABC Kit (PK-4000, Vector Laboratories) (see Suppl. methods for details). Images were acquired using an Olympus BX61 microscope or a Pannoramic 250 digital scanner (3DHISTECH, Budapest, Hungary).

For measuring percentage of 6E10-immunoreactive areas, 6E10-immunolabeled sections were thresholded and the percentage of 6E10 positive area was calculated using Fiji (v2.0.0) and the “measure” function. For measuring microglial number, Iba1 positive cells in the region of interest (ROI) were manually counted and number was normalized to the area of the ROI. ROI (PFC and hippocampus) were determined and outlined by manual tracing.

### Western blot

 30 µg of protein lysates prepared from hemibrains homogenized in RIPA buffer were loaded in Criterion XT 4-20% Bis–Tris gels and transferred onto PVDF membrane (0.45 μm; Millipore, Billerica, MA, USA). Membranes were probed with anti-C1q antibody (1:1000, cat#ab182451, Abcam) and subsequently incubated with anti-rabbit HRP-conjugated secondary antibodies (1:2000, cat#PI-1000, Vector laboratories). Normalization was achieved using GAPDH. Membranes were probed with anti-GAPDH antibody (1:5000, cat#sc32233, Cruz Biotechnology, Dallas, TX, USA) and subsequently incubated with anti-mouse HRP-conjugated secondary antibodies (1:2000, cat#PI-2000, Vector laboratories). Membranes were developed with ECL Western blotting substrate (Pierce, Rockford, IL, USA) using the Fujifilm LAS-3000 developer (Stamford, CT, USA). Integrated density of immunoreactive bands were measured using MultiGauge Software (FujiFilm) (see Suppl. methods for details).

### Aß assays and oligomer epitope characterization

Samples were processed as previously described [28, 37, 38]. Hemibrains were processed via serial detergent fractionation with ultracentrifugation to produce TBS-soluble, Triton-X-soluble, and formic-acid-soluble Aβ fractions (Suppl. Figure 6a  and Suppl. methods [39] for detailed serial detergent fractionation method). For analysis of native oligomeric Aβ protein structure, 2 μl protein samples from the TBS-soluble fraction were spotted onto activated PVDF membrane (0.22 μm; Millipore). Membranes were incubated with either rabbit pAb A11 (anti-prefibrillar oligomers; 0.5 μg/ml; gift from Charles Glabe, University of California Irvine), rabbit pAb OC (anti-fibrillar oligomers and fibrils; 0.25 μg/ml; gift from Charles Glabe) or mouse mAb NU-4 (anti-oligomers; 1 μg/ml; gift from William Klein, Northwestern University) [28, 40, 41]. Generation, purification, and characterization of A11, OC and NU-4 have been described previously [40, 41]. Membranes were incubated with appropriate anti-mouse or -rabbit HRP-conjugated secondary antibody (1:20,000; Vector laboratories) and developed as described in the western blot section. Normalization to total APP/Aβ signal was achieved by detection of human APP transgene metabolites with the anti-Aß antibody 6E10 (1:1000, Covance).

To quantify Aβ42 and Aβ40 levels, human/rat Aβ1–40/1–42 ELISA kits (Wako, Richmond, VA) were used according to the manufacturer’s instructions. Absolute concentrations of total or oligomeric Aβ were normalized to initial tissue weight.

### Field electrophysiology

Coronal brain slices containing the hippocampal formation were prepared as previously described [28, 42]. Animals were anesthetized with isoflurane, and brains were rapidly removed from the skull and placed in an ice-cold modified ACSF solution (215 mM sucrose, 2.5 mM KCl, 1.6 mM NaH_2_PO_4_, 4 mM MgSO_4_, 1 mM CaCl_2_, 4 mM MgCl_2_, 20 mM glucose, 26 mM NaHCO_3_, pH = 7.4). Coronal brain slices (400 µm thick) were prepared with a Vibratome VT1000S (Leica Microsystems) and then incubated at room temperature for ≥3 h in a physiologic ACSF (120 mM NaCl, 3.3 mM KCl, 1.2 mM Na_2_HPO_4_, 26 mM NaHCO_3_, 1.3 mM MgSO_4_, 1.8 mM CaCl_2_, 11 mM Glucose, pH = 7.4). The hemislices were transferred to a recording chamber perfused with ACSF at a flow rate of ~2 mL/min using a peristaltic pump; experiments were performed at 28.0 ± 0.1 °C. Recordings were acquired with a GeneClamp 500B amplifier (Axon Instruments) and Digidata 1440A (Molecular Devices). All signals were low-pass filtered at 2 kHz and digitized at 10 kHz. Field recordings were obtained using a patch-type pipette filled with ACSF and placed in the middle third of stratum radiatum in area CA1. Field excitatory postsynaptic potentials (fEPSPs) were evoked by activating the Shaffer collaterals with a concentric bipolar electrode placed in the middle third of stratum radiatum 150–200 µm away from the recording pipette (Fig. 5a). Square-wave current pulses (60 ms pulse width) were delivered through a constant-current stimulus isolator (Isoflex, AMPI). Input–output curves were generated by a series of stimuli ranging from 30 to 80 µA. Paired-pulse facilitation (PPF) was measured by delivering two stimuli at 20, 50 and 100 ms interstimulus intervals. Each interstimulus interval was repeated three times, and the resulting potentials were averaged. The paired-pulse ratio was calculated by dividing the slope of the second EPSP by the slope of the first EPSP. All results were analyzed by ANOVA followed by Tukey post hoc tests. Baseline recordings (stable for 20 min) were made every 30 s using stimuli that yielded a response equal to 50% of spike threshold. For long-term potentiation (LTP) and long-term depression (LTD) experiments, synaptic strength was measured by EPSP slope with stimuli that yielded a response equal to 30–40% of spike threshold, delivered every 30 s. LTP was induced after 20 min of stable baseline recording with theta burst stimulation, consisting of a series of 10 bursts of 4 stimuli (100 Hz within the burst and 200 ms interburst interval) repeated four times (10 s apart) and delivered at an intensity giving a baseline response equal to 75% of spike threshold. LTD was induced after 20 min of stable baseline recordings by bath application of (*RS*)-dihydroxyphenylglycine (DHPG; 100 µM), a selective mGluR1/mGluR5 agonist, for 5 min. fEPSPs were collected for at least 60 min during DHPG washout.

### Barnes Maze

The Barnes Maze test [43, 44] consists of a rotatable circular platform (1.22 m in diameter and 1 m from the floor) with 20 holes in periphery. A removable box was placed underneath one of the holes for escape, and visual cues were placed on the walls of the room. Mice were transported from their cages to the center of the platform via a closed starting chamber where they remained for 10 s prior to exploring the maze for 3 min. Mice failing to enter the escape box within 3 min were guided to the escape box by the experimenter, and the latency was counted as 180 s. Mice were allowed to remain in the escape box for 1 min before the next trial. Two trials per day were performed on four consecutive days. The platform and the escape box were wiped with 70% ethanol after each trial to eliminate the use of olfactory cues to locate the target hole. All trials were recorded by video camera and analyzed with ANY-maze video tracking software (Stoelting Co, Wood Dale, USA).

### Statistics

Graphs represent the mean of all samples in each group ± SEM. *n* Values and statistical tests are indicated in figure legends. Shapiro–Wilk normality tests were used. Analyses used include one-way ANOVA, two-way ANOVA, and Mann–Whitney tests. Significance is set at *p* value ≤ 0.05. No mice were excluded for RNA sequencing analysis. For biochemical, histological, behavioral, and electrophysiological analyses, outliers were detected using Grubbs’s test (extreme studentized deviate method) with *α* = 0.05. Investigators were blinded to sample identity for behavioral, electrophysiological, and histological analyses. Sequencing library and RNA sequencing were performed by the Genomics Core Facility at Icahn School of Medicine at Mount Sinai. Samples were assayed with operators blinded to sample identity (e.g., genotype).

### Data and software availability

Gene expression data have been deposited electronically to the Synapse Web Portal (https://www.synapse.org, accession number syn10378730) in accordance with data sharing policies established by the NIH AMP-AD consortium. Specific software will also be made available upon request.

## Results

In order to validate in vivo in mice the driver role of *TYROBP* in sporadic LOAD and to demonstrate that the manipulation of TYROBP level leads to molecular changes observed human LOAD, we performed a battery of molecular, behavioral, electrophysiological analyses, and generated transcriptomic profiles in 8-month-old WT and *APP/PSEN1* mice that were either WT, heterozygous- or homozygous-null for *Tyrobp* (Suppl. Figure 1).

### *TYROBP* is upregulated in human sporadic LOAD postmortem samples

We evaluated *TYROBP* gene expression at different stages of LOAD in a large-scale postmortem brain transcriptomic dataset from the Mount Sinai Brain Bank (MSBB) AD cohort [6, 35]. In all four brain regions profiled with RNA-sequencing (RNA-seq) from this cohort, *TYROBP* mRNA was upregulated by at least 1.2-fold in the demented subjects compared to the nondemented controls (*p* < 0.05) (Suppl. Figure 2). We confirmed the disease-associated upregulation of *TYROBP* in brains in a second large postmortem brain RNA-seq dataset from the ROSMAP AD cohort (1.1-fold, *p* = 0.02) (data not shown).

### In an *APP/PSEN1* mouse, constitutive absence of TYROBP prevents the expression of pro-inflammatory and microglial sporadic LOAD-associated genes

**Fig. 1:**
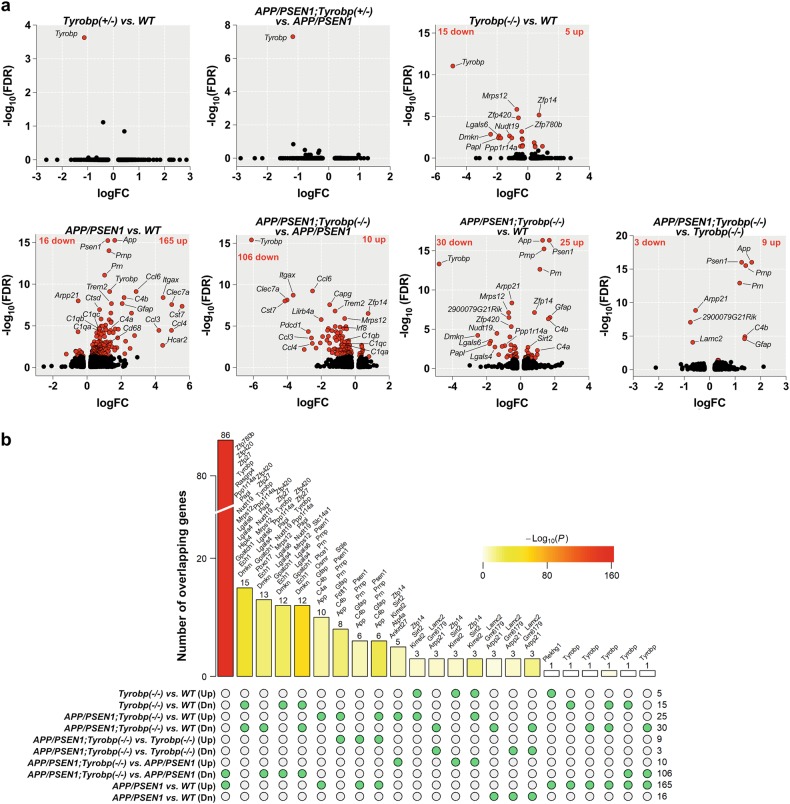
RNA sequencing and differentially expressed genes (DEGs) in mice. The absence of TYROBP downregulated half of the genes upregulated in *APP/PSEN1* mice vs. WT. RNA sequencing was performed on the prefrontal cortex for six groups of mice at 8-month -old of age (WT (*n* = 4), *Tyrobp*^+/−^ (*n* = 3), *Tyrobp*^−/−^ (*n* = 4), *APP/PSEN1* (*n* = 4), *APP/PSEN1;Tyrobp*^+/−^ (*n* = 4), and *APP/PSEN1;Tyrobp*^−/−^ (*n* = 5)). **a** Volcano plot representations of the DEGs in WT and *APP/PSEN1* mice heterozygous, knockout or WT for *Tyrobp*. Red dots represent DEGs at an FDR of 0.05. **b** Comparison of the shared and unique DEGs (FDR < 0.05) in *APP/PSEN1*, *APP/PSEN1;Tyrobp*^−/−^ and *Tyrobp*^−/−^ mice. The bar height denotes the number of DEGs overlapping in a given comparison as specified by the green circles underneath. The *p* value denotes the significance of the overlap size. List of overlapping DEGs are shown above the bars except for the comparison between *APP/PSEN1;Tyrobp*^−/−^ vs. *APP/PSEN1* (Dn) and *APP/PSEN1;Tyrobp*^−/−^ vs. WT (see Suppl. Figure 3c). Up upregulated; Dn downregulated genes

We generated transcriptomic profiles of 24 PFC samples from 8-month-old male WT and *APP/PSEN1* mice that were either WT, or heterozygous- or homozygous-null for *Tyrobp* (*n* = 3–5 samples per group). At an FDR of 0.05, *Tyrobp* was the only differentially expressed gene (DEG) in heterozygous-null mice in comparison to WT and *APP/PSEN1* mice, indicating that in a WT or pathological (*APP/PSEN1*) background, deletion of one allele of *Tyrobp* does not induce a significant transcriptomic change at a 5% FDR (Fig. 1a). The *Tyrobp* homozygous-null transcriptome contained 20 DEGs in comparison to WT mice. Among them, 5 were upregulated and 15 were downregulated (Fig. 1a). These results are consistent with our previous report in 4-month-old mice showing that deficiency or absence of TYROBP in a WT background without immune stimulation perturbs expression of only a limited number of genes [28] (Suppl. Figure 3a).

We next evaluated the changes of the transcriptome in the *APP/PSEN1* mouse model and identified 181 DEGs in comparison to WT (Fig. 1a). Over 90% of the identified DEGs were upregulated (165 upregulated and 16 downregulated genes). Among the most significantly upregulated genes, we observed several LOAD-associated genes, including *Trem2*, *Tyrobp*, *C1q*, *Itgax* (aka *CD11c*), *Cst7*, *Clec7a*, and *Gfap* (Fig. 1a, see Suppl. Table 1 for full DEGs results) highlighting the induction of immune response components in the 8-month-old *APP/PSEN1* mice.

To evaluate the effect of the absence of TYROBP on the transcriptome of the *APP/PSEN1* mice, we compared the transcriptomes of *APP/PSEN1* mice homozygous-null for *Tyrobp* against *APP/PSEN1* mice with homozygous WT *Tyrobp* and identified 116 DEGs (Fig. 1a). Contrary to the above comparison (*APP/PSEN1* vs. WT), the majority of DEGs were downregulated (10 upregulated and 106 downregulated genes). Notably, there was a decrease in the expression of *Trem2*, multiple complement-related genes (*C1qa*, *C1qb*, *C1qc*, and *Itgax*), *Cst7*, and several chemokines (*Ccl4*, *Ccl6*, *Ccl9*, and *Ccl3l3*) (Fig. 1a, Suppl. Table 1).

We calculated the proportion of genes that were shared in the different comparisons (Fig. 1b). There were 86 shared genes between the comparisons *APP/PSEN1* vs. WT and *APP/PSEN1;Tyrobp*^−/−^ vs. *APP/PSEN1* [Fisher’s exact test (FET) *p* = 6.6e−161, 76.1-fold enrichment (FE)]. Strikingly, these genes were upregulated in the brains of cerebral amyloidosis mice and downregulated in those of cerebral amyloidosis mice with *Tyrobp* KO (Fig. 1b, Suppl. Figure 3c). The 86 genes included a number of key genes involved in the switch from homeostatic microglia to disease-associated microglia (DAM), including *Trem2*, *Itgax*, *Ctsd*, *Cst7*, *Clec7a*, *Ccl6*, *Cd68*, and *Selplg* [45] (Suppl. Figure 3c). In addition, the absence of TYROBP in the *APP/PSEN1* background also decreased the expression of *Irf8* and *Irf5*, two important transcription factors regulating pro-inflammatory cytokine expression [46, 47].

Given the apparent decreased expression of nearly 50% of the upregulated DEGs associated with the *APP/PSEN1* mice, we next compared the transcriptome of *APP/PSEN1;Tyrobp*^−/−^ mice vs. WT mice. We identified 55 DEGs (25 up and 30 down), substantially fewer than the 181 DEGs between *APP/PSEN1* vs. WT (Fig. 1a). Notably, expression of several chemokines, transcription factors, and DAM genes increased in *APP/PSEN1* mice vs. WT were normalized in *APP/PSEN1;Tyrobp*^−/−^ mice vs. WT (Fig. 1a, Suppl. Table 1). Accordingly, comparison of the transcriptome of *APP/PSEN1;Tyrobp*^−/−^ mice vs. *Tyrobp*^−/−^ mice reported a limited number of DEGs (12 DEGs; 9 up; and 3 down) (Fig. 1a, Suppl. Table 1). While the absence of TYROBP in the *APP/PSEN1* did not normalize the expression of the astrocytic genes *C4b* and *Gfap vs*. WT or *Tyrobp*^−/−^, our data suggest that the absence of TYROBP in the *APP/PSEN1* mouse model substantially decreases the induction of the pro-inflammatory immune response observed in the brains of *APP/PSEN1* mice.

### Constitutive absence of TYROBP prevents dysregulation of the major AD-related complement transcriptomic subnetwork

**Fig. 2:**
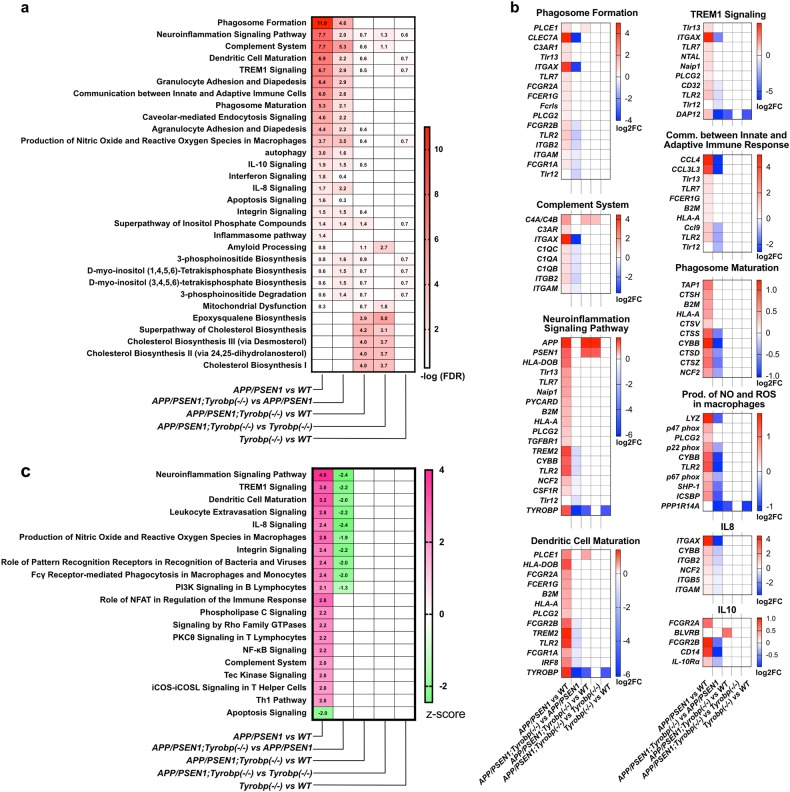
Gene set enrichment analysis on the DEGs of *Tyrobp*^−/−^, *APP/PSEN1;Tyrobp*^−/−^ and *APP/PSEN1* mice at 8-months old using ingenuity pathway analysis (IPA). The absence of TYROBP in *APP/PSEN1* mice alleviated the induction of the immune/pro-inflammatory response observed in the *APP/PSEN1* mouse brain. **a** Heatmap of selected dysregulated canonical pathways in *APP/PSEN1*, *APP/PSEN1;Tyrobp*^−/−^, and *Tyrobp*^−/−^ mice and in the different comparisons. Data presented as −log(FDR). Pathways were selected for known or suspected relevance to AD pathophysiology. Full results of canonical pathway analyses are presented in Suppl. Table 2. **b** Heatmap of significantly affected genes in selected affected canonical pathways. Data presented as −log2 (fold change). Full results are presented in Suppl. Table 2. **c** Heatmap of predicted activated or inhibited canonical pathways in *APP/PSEN1*, *APP/PSEN1;Tyrobp*^−/−^ and *Tyrobp*^−/−^ mice and in the different comparisons. Data presented as *z*-score

To identify biological pathways that may be dysregulated in WT and *APP/PSEN1* mice WT or KO for  *Tyrobp*, we performed gene set enrichment analysis (GSEA) using IPA (Fig. 2). We first sought to identify the pathways that were specifically dysregulated in the 8-month-old amyloidosis mouse model and performed GSEA on the list of genes that were differentially expressed in the *APP/PSEN1* mice in comparison to WT mice (FDR < 0.05). At −log(FDR) > 1.3, 56 canonical pathways were dysregulated in the *APP/PSEN1* vs. WT mice, several of which were associated with immune response and glial cell activation (Fig. 2a for selected canonical pathways, see Suppl. Table 2 for full results of canonical pathway analyses). Top dysregulated pathways included phagosome formation and maturation, neuroinflammation signaling, complement system, dendritic cell maturation, production of nitric oxide (NO) and reactive oxygen species (ROS), and IL-10 and IL-8 signaling. A list of DEGs associated with selected dysregulated pathways is presented in Fig. 2b. Prediction of the activation state revealed activation of several pathways including neuroinflammation signaling, TREM1 signaling, production of NO and ROS, and complement system in the *APP/PSEN1* mice vs. WT mice (−log(FDR) > 1.3 and *z*-score > 2) (Fig. 2c, Suppl. Table 2). The apoptosis signaling pathway was predicted to be decreased in the *APP/PSEN1* mice (−log(FDR) > 1.3 and *z*-score < −2). This pathway was driven by an increased expression of antiapoptotic genes (*Naip3*, *Naip5*, and *Bcl2a1b* and *d*) implicated in inflammasome activation or upregulated during inflammatory processes [48, 49].

We next performed GSEA on the list of DEGs in *APP/PSEN1;Tyrobp*^−/−^ vs. *APP/PSEN1* mice (FDR < 0.05). Thirty-two canonical pathways were dysregulated (−log(FDR) > 1.3). This number is substantially higher than our previous report in *APP/PSEN1;Tyrobp*^−/−^ vs. *APP/PSEN1* mice at 4-month-old (Suppl. Figure 3b). Complement system, phagosome formation and production of NO and ROS were the most significantly dysregulated in the *APP/PSEN1* KO for *Tyrobp* vs. *APP/PSEN1* WT for *Tyrobp* (−log(FDR) = 5.32, 4.58, and 3.45, respectively). Contrary to the previous comparison, prediction of the activation state showed down-regulation of pathways associated with immune response and glial cell activation (e.g., neuroinflammation signaling pathway, Trem1 signaling, dendritic cell maturation, IL-8 signaling, and production of NO and ROS in macrophages) (−log(FDR) > 1.3 and *z*-score  ≤−2) (Fig. 2c, Suppl. Table 2).

We analyzed whether these apparent beneficial effects of the absence of TYROBP on the cerebral amyloidosis transcriptome could decrease the number of perturbed canonical pathways observed in the *APP/PSEN1* mice vs. WT. Only seven pathways were significantly dysregulated in *APP/PSEN1;Tyrobp*^−/−^ vs. WT (−log(FDR) > 1.3), five of them being related to cholesterol metabolism (superpathway of cholesterol biosynthesis, cholesterol biosynthesis I–III, and epoxysqualene biosynthesis) and two of the dysregulated pathways were involved in inositol biosynthesis and metabolism (d-myo-inositol (1,4,5)-trisphosphate biosynthesis, superpathway of inositol phosphate compounds) (Fig. 2a, Suppl. Table 2). Importantly, all canonical pathways activated in the brains of *APP/PSEN1* mice vs. WT (*z*-score > 2) were normalized in *APP/PSEN1;Tyrobp*^−/−^ vs. WT (Fig. 2c). GSEA in the *APP/PSEN1;Tyrobp*^−/−^ mice vs. *Tyrobp*^−/−^ mice reported nine dysregulated pathways. However, considering the limited number of DEGs in this comparison (12 DEGs), the importance of the dysregulations is unclear. No canonical pathways were significantly dysregulated in the *Tyrobp*^−/−^ vs. WT mice.

These results demonstrate that the absence of TYROBP reduces the perturbations of the transcriptome observed in the brains of *APP/PSEN1* mice and alleviates the induction of the immune/pro-inflammatory response observed in the *APP/PSEN1* mouse brain.

### Absence of TYROBP decreased the induction of C1q expression in *APP/PSEN1*

The complement system plays an important role in the pathogenesis of AD [26, 27, 50]. In our transcriptomic analysis, absence of TYROBP in *APP/PSEN1* mice alleviates the increased expression of several central complement components associated with *APP* and/or *PSEN1* mutations. We assessed by qPCR, western blot and immunostaining the expression of C1q, the initiating protein of the classical complement cascade, in male and female *APP/PSEN1* mice WT or null for *Tyrobp* at 8 months of age (Suppl. Figure 4). Consistent with the RNAseq results, mRNA level of C1q was increased in the *APP/PSEN1* mice as compare to WT mice and was normalized in *APP/PSEN1;Tyrobp*^−/−^ (Suppl. Figure 4a). Western blot and immunostaining confirmed the decreased C1q expression in *APP/PSEN1;Tyrobp*^−/−^ mice as compared to that of *APP/PSEN1* mice (Suppl. Figure 4b–d).

### Brain network signatures in *APP/PSEN1* mice are enriched for human sporadic LOAD network signatures

We intersected the *APP/PSEN1* mouse signature (*APP/PSEN1* vs. WT) with multiple LOAD brain gene expression signatures previously identified in human brains. Suppl. Table 3 summarizes the source of the human LOAD gene signatures and the intersection analysis results obtained from the FET. Genes upregulated in the brains of *APP/PSEN1* mice were significantly enriched for genes upregulated in CA1 of the hippocampus (4.1-fold, adjusted *p* value 6.1 × 10^−4^) or CA3 (3.3-fold, adjusted *p* value 8.1 × 10^−4^) of LOAD brains as identified by Miller et al. [51]. Meanwhile, genes upregulated in *APP/PSEN1* mouse brains were also significantly enriched for genes positively correlated with the Braak score in the PFC region of the Harvard Brain Tissue Resource Center (HBTRC) cohort (2.4-fold, adjusted *p* value 1.6 × 10^−3^) [6]. The genes downregulated in the brains of *APP/PSEN1* mice were not enriched in human AD signatures, perhaps at least partially attributable to the limited number of downregulated genes identified in the current study.

### *TYROBP* knockout signatures are enriched in *TYROBP* regulated networks in human LOAD brains

**Fig. 3:**
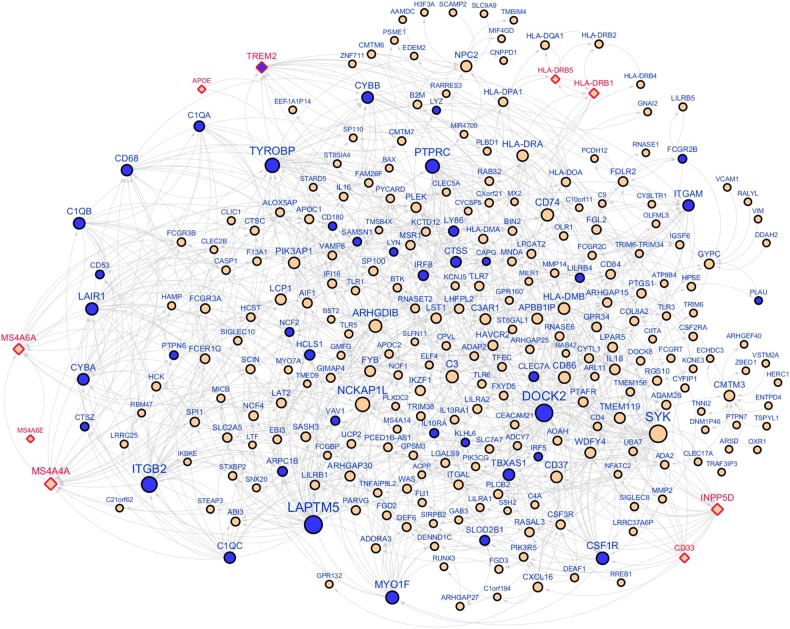
An immune/microglia enriched gene network regulated by *TYROBP*. The Bayesian causal network, comprised of the three-layer neighborhoods of *TYROBP* (716 genes), was significantly enriched (10.3-fold, FET *p* value 2.7 × 10^−51^) for the genes downregulated (blue color) by *Tyrobp* knockout in *APP/PSEN1* mice. Diamond shape denotes AD GWAS hits

*TYROBP* was identified as a network key driver implicated in LOAD based on the Bayesian causal regulatory network analysis in the HBTRC expression dataset [5]. We overlaid the *Tyrobp-*null signature detected in *APP/PSEN1* mice (*APP/PSEN1;Tyrobp*^−/−^ vs. *APP/PSEN1*) onto the HBTRC Bayesian network and found enrichment of downregulated genes in the first four layers downstream of *TYROBP* (up to 29.4-fold; FET *p* value 3.5 × 10^−^^7^–1.2 × 10^−33^), thus validating the *TYROBP*-centered regulatory network (Fig. 3). One key output of the NIH AMP-AD program has been RNA-seq datasets from over 1000 human brain tissues across multiple brain regions, generated from individual LOAD cohorts, including the aforementioned MSBB and ROSMAP. This allowed us to build new Bayesian networks from each individual brain region and combine them with the HBTRC networks to create a more comprehensive causal network, termed union Bayesian network (see Suppl. Text). In this union Bayesian network, we observed a stronger enrichment of downregulated genes by deletion of *Tyrobp* in the first four layers of the network neighborhood of *TYROBP* (FET *p* value 3.6 × 10^−15^, 5.0 × 10^−48^, 2.7 × 10^−51^, and 7.7 × 10^−23^ accordingly). For example, in the first-layer neighborhood of *TYROBP*, there was a 70-fold enrichment and one third of the 27 first-layer neighbor genes were downregulated by the deficit of TYROBP in *APP/PSEN1* mice (FET *p* value 3.6 × 10^−15^). Figure 3 shows the network topology of the up to 3-layer network neighborhood around *TYROBP*. The validated network genes included *TREM2*, a prominent LOAD GWAS gene, and complement component subunits including*C1QA* and *C1QB*. In addition to *TREM2*, we found 5 other LOAD GWAS hits in this subnetwork including *APOE*, the *MS4A* locus, *CD33*, *INPP5D*, and the *HLA* locus. Unlike *TREM2*, the expression of these GWAS genes was not altered by deletion of *Tyrobp*. As expected, the upregulated genes in the brains of *APP/PSEN1* mice were also significantly enriched in the neighbors of *TYROBP* (9.9-fold, FET *p* value 4.3 × 10^−71^; Suppl. Figure 5). In summary, this causal network analysis shows that the human LOAD causal networks are highly predictive of the *Tyrobp-*null murine signature.

We further intersected the present mouse gene signatures with co-expression network modules identified across multiple human LOAD datasets. As summarized in Suppl. Table 4, the genes upregulated in *APP/PSEN1* mice were significantly enriched in human immune response modules. Meanwhile, *Tyrobp*^−/−^ signatures in *APP/PSEN1* mice were also enriched in the same human immune response modules. Many of these immune response modules were highly associated with AD pathology, including the top module “yellow” in the PFC co-expression network from the HBTRC data in which *TYROBP* was a module member [5]. The result suggests that *Tyrobp* modulates the immune response pathways which are activated by transgenic expression of *APP/PSEN1*.

### Constitutive deletion of *Tyrobp* does not alter amyloid burden in 8-month-old *APP/PSEN1* mice

**Fig. 4:**
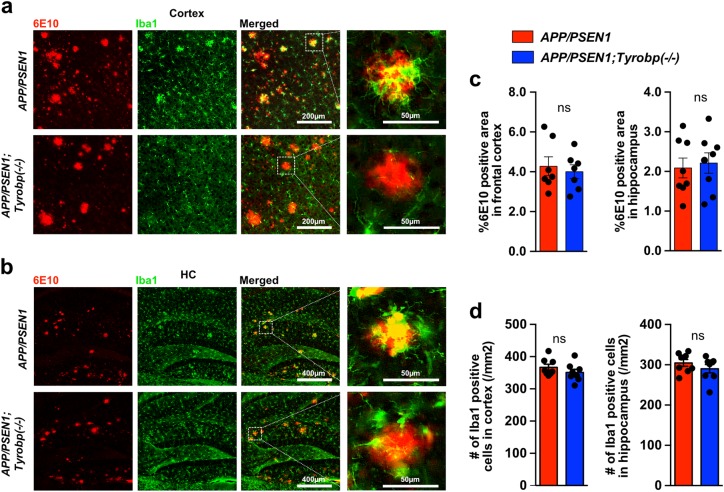
Absence of TYROBP does not modify the percentage of 6E10 immunoreactive Aß deposits nor the total number of microglia in 8-month -old *APP/PSEN1* mice. **a**, **b** Images of 6E10-immunoreactive plaques (red) and anti Iba1-immunostained microglia (green) in PFCs (**a**) and hippocampi (HC) (**b**) of *APP/PSEN1* and *APP/PSEN1;Tyrobp*^−/−^ mice. **c** Percentage of 6E10-immunoreactive area in PFC and hippocampi of male and female *APP/PSEN1* (*n* = 7–8), and *APP/PSEN1;Tyrobp*^−/−^ (*n* = 7–8) mice. Sex effect: ns. **d** Quantification of the number of Iba1-immunostained microglia in cortices and hippocampi of male and female *APP/PSEN1* (*n* = 8) and *APP/PSEN1;Tyrobp*^−/−^ (*n* = 8) mice. Sex effect: ns. Mann–Whitney test was for statistical comparisons. ns not significant. Data presented as mean ± SEM

We assessed whether the absence of TYROBP would modulate Aß deposition in the PFC and hippocampus (HC) of male and female *APP/PSEN1* mice. The area covered by 6E10-immunoreactive plaques was unchanged by the deletion of *Tyrobp* in *APP/PSEN1* mice (Fig. 4a–c). We and others previously reported decreased microglial clustering around the Aß plaques in *Tyrobp*^−/−^ mice with Aß pathology [28, 52]. We observed this same phenomenon in the 8-month-old *APP/PSEN1;Tyrobp*^−/−^ as compared to *APP/PSEN1* mice with normal levels of TYROBP (Fig. 4a, b). We then asked whether reduced clustering of microglia around plaques in the *APP/PSEN1* mice null for *Tyrobp* was due to a reduction in the total number of brain microglia, as observed in *Trem2*^−/−^ mice with Aß pathology [52, 53]. Iba1 immunostaining in 8-month-old *APP/PSEN1* mice null for *Tyrobp* did not show differences in the total number of microglia in cortex or hippocampus as compared to *APP/PSEN1* mice with normal levels of TYROBP (Fig. 4d).

### Constitutive absence of TYROBP is associated with elevated levels of soluble Aß but reduced levels of insoluble Aß in 8-month-old *APP/PSEN1* mice

We assessed whether the absence of TYROBP would modulate levels of Aß species in *APP/PSEN1* mice. We measured levels of Aß40 and Aß42 in TBS, Triton-X, and formic-acid soluble Aß fractions from brains of 8-month-old male and female *APP/PSEN1* mice WT or null for *Tyrobp* (Suppl. Figure 6a). In both males and females, absence of TYROBP was associated with increased levels of Aß40 and Aß42 in the most soluble fraction (TBS) (Suppl. Figure 6b,c). While not statistically significant, a trend toward a decreased Aß42/40 ratio was observed in the *APP/PSEN1;Tyrobp*^−/−^ mice as compared to *APP/PSEN1* mice. This effect was more obvious in females than in males (*p* value = 0.0635 and *p* value = 0.2587, respectively) (Suppl. Figure 6d). Conversely, levels of both Aß40 and Aß42 were reduced in the Triton-X fraction as compared to *APP/PSEN1* mice with normal levels of TYROBP (Suppl. Figure 6e, f) without any change in the Aß42/40 ratio (Suppl. Figure 6g). The levels of Aß40 and Aß42 did not change in the formic-acid fraction (Suppl. Figure 6h,i), but Aß40 levels trended lower in *APP/PSEN1;Tyrobp*^−/−^ mice as compared to sex-matched *APP/PSEN1* mice leading to an increased Aß42/40 ratio in the *APP/PSEN1;Tyrobp*^−/−^ mice. Notably and in accordance with our previous results [28, 54], female *APP/PSEN1* mice WT or null for *Tyrobp* had higher levels of Aß40 and 42 in the formic-acid fractions when compared to genotype-matched males.

We next assayed oligomeric Aß peptides using antibodies NU-4, A11, and OC antibodies to distinguish among Aß conformers (Suppl. Figure 6k–m), which have been correlated with impaired cognitive performances in humans and mice [55]. At 8 months of age, the absence of TYROBP played no obvious role in determining levels of NU-4-, A11-, or OC-epitope-containing oligomeric Aß.

### Deletion of *Tyrobp* ameliorates aberrant synaptic plasticity in *APP/PSEN1* mouse hippocampus

**Fig. 5:**
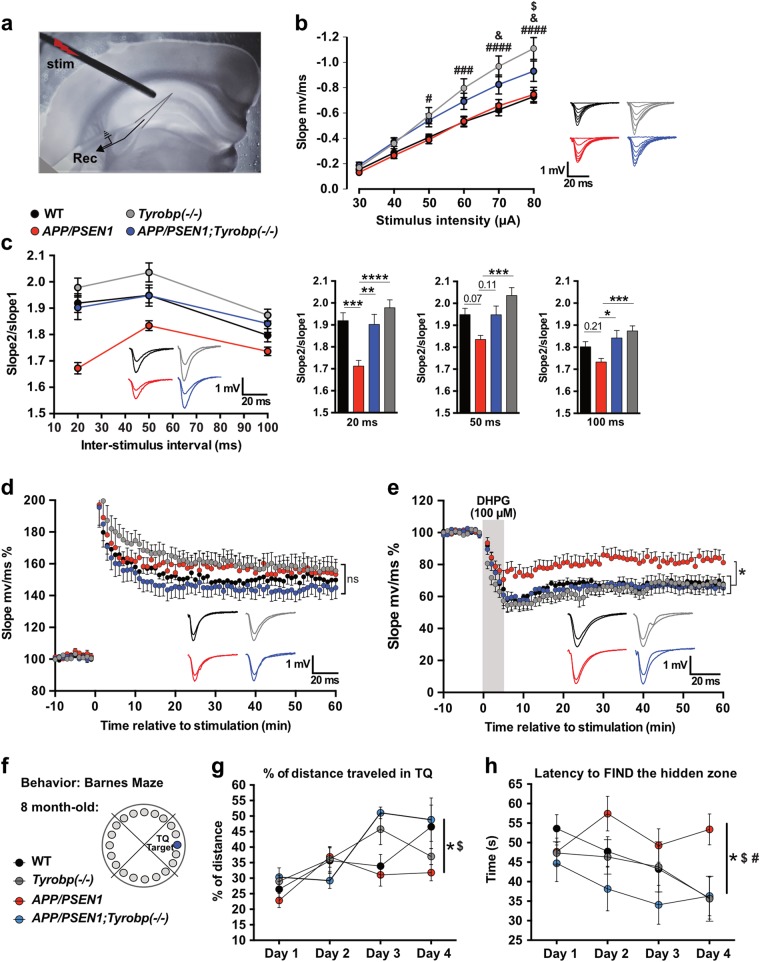
Constitutive deletion of *Tyrobp* normalizes altered synaptic plasticity and prevents defects in spatial learning behavior in 8-month-old *APP/PSEN1* mice. **a** Electrode placement for field recordings of synaptic potentials. A bipolar stimulating electrode (“stim”) was positioned above the Schaffer collaterals in area CA3, 150–200 µm lateral to the recording electrode (Rec) in stratum radiatum of area CA1. **b** Basal input–output relationship for fEPSPs in 8-month-old WT (*n* = 8 mice; 23 recordings), *Tyrobp*^−/−^ (*n* = 6 mice; 22 recordings), *APP/PSEN1* (*n* = 5 mice; 16 recordings), and *APP/PSEN1;Tyrobp*^−/−^ (*n* = 6 mice; 17 recordings) mice. Two-way ANOVA corrected for multiple comparisons (Tukey) was used for statistical comparisons. ^#^*p* < 0.05; ^###^*p* < 0.001; ^####^*p* < 0.0001 in *Tyrobp*^−/−^ vs. WT and *Tyrobp*^−/−^ vs. *APP/PSEN1*. ^&^*p* < 0.05 in *APP/PSEN1;Tyrobp*^−/−^ vs. WT. ^$^*p* < 0.05 in *APP/PSEN1;Tyrobp*^−/−^ vs. *Tyrobp*^−/−^. **c** Left panel: paired-pulse facilitation (PPF) in 8-month-old WT (*n* = 8 mice; 24 recordings), *Tyrobp*^−/−^ (*n* = 6 mice; 23 recordings), *APP/PSEN1* (*n* = 5 mice; 18 recordings), and *APP/PSEN1;Tyrobp*^−/−^ (*n* = 6 mice; 18 recordings) mice. Right panel: summary of PPF data. One-way ANOVA corrected for multiple comparisons (Tukey) was used for statistical comparisons. *p < 0.05, **p < 0.01; ***p < 0.001; ****p < 0.0001. **d** Synaptically induced long-term potentiation (LTP) in WT (*n* = 7 mice; 11 recordings), *Tyrobp*^−/−^ (*n* = 6 mice; 12 recordings), *APP/PSEN1* (*n* = 5 mice; 9 recordings), and *APP/PSEN1;Tyrobp*^−/−^ (*n* = 6 mice; 9 recordings) mice. Two-way ANOVA corrected for multiple comparisons (Tukey) was used for statistical analysis over the final 5 min of recordings. **e** 3,5-dihydroxyphenylglyine (DHPG) induced long-term depression (LTD) in WT (*n* = 5 mice; 8 recordings), *Tyrobp*^−/−^ (*n* = 6 mice; 11 recordings), *APP/PSEN1* (*n* = 5 mice; 10 recordings), and *APP/PSEN1;Tyrobp*^−/−^ (*n* = 5 mice; 9 recordings) mice. Two-way ANOVA corrected for multiple comparisons (Tukey) was used for statistical analysis over the final 5 min of recordings. *p < 0.05. Data presented as mean ± SEM. **f** Spatial learning behavior in the Barnes Maze test in 8-month-old *APP/PSEN1* mice. Four groups of 8-month-old mice were used: WT (*n* = 9), *Tyrobp*^−/−^ (*n* = 12), *APP/PSEN1* (*n* = 17), or *APP/PSEN1;Tyrobp*^−/−^ (*n* = 6). **g** Percentage of distance traveled in the target quadrant (TQ). **h** Latency (in seconds) to find the hidden zone. One-way ANOVA on the day 4 was used for statistical comparisons, *p < 0.05 in *APP/PSEN1* vs. WT; ^$^*p* < 0.05 in *APP/PSEN1* vs. *APP/PSEN1;Tyrobp*^−/−^*;* ^#^*p* < 0.01 in *APP/PSEN1* vs. *Tyrobp*^−/−^. Data presented as mean ± SEM

Synaptic plasticity is abnormal in the hippocampus of *APP/PSEN1* mice [56], and in 4-month-old mice, we found that *Tyrobp* deletion partially rescued the normal synaptic phenotype [28]. In hippocampal slices from 8-month-old mice, basal transmission at CA3-CA1 synapses was significantly enhanced in *Tyrobp*^−/−^ and *APP/PSEN1;Tyrobp*^−/−^ mice as compared to WT controls, while *APP/PSEN1* mice did not differ from WT controls (Fig. 5b). We examined a short-term form of presynaptic plasticity, PPF, which is inversely related to glutamate release probability [57]. At 4 months, PPF was depressed in *APP/PSEN1* mice and was fully rescued by deletion of *Tyrobp* [28]. At 8 months, PPF was depressed in *APP/PSEN1* mice at an interpulse interval of 20 ms and was normalized by deletion of *Tyrobp* (Fig. 5c). Absence of TYROBP in WT background did not affect the PFF. We also studied two long-term forms of synaptic plasticity: synaptically induced LTP, and DHPG-induced LTD. LTP was similar in the four genotypes [WT, *Tyrobp*^−/−^, *APP/PSEN1*, and *APP/PSEN1;Tyrobp*^−/−^] (Fig. 5d). However, LTD was markedly reduced in slices from *APP/PSEN1* mice, and this deficit was fully rescued by deletion of *Tyrobp* (Fig. 5e). Of note, absence of TYROBP in WT background did not affect LTD. These results establish that as late as 8 months of age, deletion of mouse *Tyrobp* prevents the development of electrophysiological abnormalities that are present in a model of cerebral amyloidosis.

### Constitutive deletion of *Tyrobp* prevents impaired performance in Barnes maze learning and memory in *APP/PSEN1* mice

We previously concluded that deletion of *Tyrobp* can slow or delay the progression of learning deficits in *APP/PSEN1* transgenic mice during the early stages of amyloid deposition [28]. In the current study, we probed the effect of the absence of TYROBP on spatial learning and memory using the Barnes Maze Test in 8-month-old WT, *Tyrobp*^−/−^, *APP/PSEN1*, and *APP/PSEN1;Tyrobp*^−/−^ mice (Fig. 5f). The percentage of distance traveled in the target quadrant (TQ) and the latency to find the hidden zone were identical between *Tyrobp*^−/−^ mice and WT mice suggesting that deficiency of TYROBP did not have a deleterious effect on learning and memory in a WT background. *APP/PSEN1* mice showed impairment of learning and memory relative to WT mice with a shorter distance traveled in the TQ and increased latency to find the hidden zone as compare to WT mice (Fig. 5g, h). Absence of TYROBP in *APP/PSEN1* mice was associated with improved learning and memory relative to *APP/PSEN1* with WT TYROBP levels. Thus, percentage of distance traveled in the TQ and latency to find the hidden zone were similar in *APP/PSEN1;Tyrobp*^−/−^ and WT littermates. These data are consistent with a beneficial effect of *Tyrobp* deletion on learning and memory deficits associated with *APP/PSEN1* mutations.

## Discussion

Despite decades of intensive research, the pathogenesis of LOAD remains elusive, and therapeutic interventions focused on reduction of Aß accumulation have failed to provide meaningful clinical benefits. Multiscale network modeling of large-scale functional genomics data creates novel opportunities to identify key targets causally linked to LOAD, thereby offering new insights for drug-discovery programs. We previously constructed a molecular network based on whole-genome gene-expression profiling and genotyping data on 1647 autopsied brain tissues from LOAD and nondemented subjects and reported the first LOAD causal network centered on *TYROBP* [5]. In the computational model, *TYROBP* lies at the center of the complement system subnetwork, and, based on its predicted role as a hub and driver of the complement subnetwork, we predicted that changes in the level of TYROBP would lead to similar changes in the expression level of genes in that network.

Herein, we report the first biological validation of the *TYROBP*-centered LOAD network in the amyloidogenic *APP/PSEN1* mouse model. Our data indicate that constitutive deletion of *Tyrobp* prevents behavioral and electrophysiological deficits in 8-month-old Aß-amyloid-depositing mouse. These results are associated with a recapitulation of the expected complement system subnetwork in *APP/PSEN1* mice, followed by its reversal in the absence of TYROBP, predicted from the multiscale gene networks of human postmortem LOAD brain. *Tyrobp* deletion in the *APP/PSEN1* mice repressed the induction of inflammatory cytokines and numerous other genes that are upregulated in the switch from homeostatic microglia to disease-associated microglia (DAM) [45], including *Trem2*, *Clec7a*, *Cst7*, *Itgax*, and *Ctsd*. DAM are found in AD at the proximity of the Aß plaques and express genes associated with lysosomal-phagocytic, lipid metabolism, and AD risk factors including *Apoe* and *Trem2*. While we did not detect differences in *ApoE* expression, the decreased expression of numerous DAM genes highlights the role of TYROBP as an important early activator of DAM. Moreover, the absence of *APP/PSEN1* associated behavioral and electrophysiological impairments in the absence of TYROBP suggests that at least in this mouse model, decreasing DAM activation might play a beneficial role in the progression of AD.

The complement system is robustly activated in our dataset. This system plays a crucial role in physiologic synaptic pruning [50], is activated in human and mouse AD brains [27, 58–60] and is associated with early synapse loss in AD [26, 27]. Amyloidogenic *APP/PSEN1* mouse models with reduced components of the complement system, e.g., C3, C1q, or CR3, showed improved performance on learning and memory tasks despite having increased Aß deposition [26]. Similarly, herein and in our previous report [28], we show that deletion of *Tyrobp* in the *APP/PSEN1* mouse model did not play a prominent role in Aß deposition but reversed the behavioral and electrophysiological alterations associated with the *APP/PSEN1* mutations. We also observed that deletion of *Tyrobp* in a tauopathy mouse model reduced the expression of C1q and improved learning behavior and synaptic function despite a paradoxical increased in the spread and the phosphorylation state of tau [61]. The regulation of the complement subnetwork by TYROBP, including a decrease in several of the same components previously reported further supports a beneficial effect of decreased complement pathway activation in AD pathology. Notably, *APP/PSEN1* mice with deletion of *Progranulin* (*Grn*) demonstrate overexpression of some *TYROBP* network genes, including C1q [62], and while they have reduced diffuse Aß and axonal dystrophy with partially improved behavioral deficits, they also manifest increased neuronal injury. We report that deletion of *Tyrobp* in the *APP/PSEN1* model also reduces *Grn* expression level in *APP/PSEN1* alone.

This study represents the first in vivo validation of a hub/driver gene and subnetwork predicted by integrative network analyses of human postmortem sporadic LOAD brain and suggests that reduction of TYROBP level or inhibition of its activity could represent a potential therapeutic target for AD prevention and/or treatment. Moreover, this study validates the multiscale network for identifying gene subnetworks and key drivers in complex diseases and for providing new insights for drug discovery programs.

## Electronic supplementary material

Supplementary Legends

Supplementary Figure 1

Supplementary Figure 2

Supplementary Figure 3

Supplementary Figure 4

Supplementary Figure 5

Supplementary Figure 6

Supplementary Table 1

Supplementary Table 2

Supplementary Table 3

Supplementary Table 4

Supplementary Methods
