# Correction: Integrative approach to sporadic Alzheimer’s disease: deficiency of TYROBP in cerebral Aβ amyloidosis mouse normalizes clinical phenotype and complement subnetwork molecular pathology without reducing Aβ burden

**DOI:** 10.1038/s41380-018-0301-4

**Published:** 2018-11-21

**Authors:** Jean-Vianney Haure-Mirande, Minghui Wang, Mickael Audrain, Tomas Fanutza, Soong Ho Kim, Szilvia Heja, Ben Readhead, Joel T. Dudley, Robert D. Blitzer, Eric E. Schadt, Bin Zhang, Sam Gandy, Michelle E. Ehrlich

**Affiliations:** 1 Department of Neurology, Icahn School of Medicine at Mount Sinai, New York, NY 10029 USA; 2 Department of Genetics and Genomic Sciences and Icahn Institute of Genomic Sciences, Icahn School of Medicine at Mount Sinai, New York, NY 10029 USA; 3 Departments of Pharmacological Sciences and Psychiatry, Icahn School of Medicine at Mount Sinai, New York, NY 10029 USA; 4 Sema4, a Mount Sinai venture, Stamford, CT 06902 USA; 5 Department of Psychiatry and Alzheimer’s Disease Research Center, Icahn School of Medicine at Mount Sinai, New York, NY 10029 USA; 6 Department of Pediatrics, Icahn School of Medicine at Mount Sinai, New York, NY 10029 USA

Correction to: *Molecular Psychiatry*; 10.1038/s41380-018-0255-6; published online 03 October 2018.

This article was originally published under standard licence, but has now been made available under a CC BY 4.0 license. The PDF and HTML versions of the paper have been modified accordingly.

