# Supplementary material for: Integrative approach to sporadic Alzheimer’s disease: deficiency of TYROBP in cerebral Aβ amyloidosis mouse normalizes clinical phenotype and complement subnetwork molecular pathology without reducing Aβ burden
Source: Mol Psychiatry. 2018 Oct 3;24(3):431–46. doi: 10.1038/s41380-018-0255-6 (PMC6494440; doi:10.1038/s41380-018-0255-6)
Supplement: Supplementary file 1 — Supplementary Legends [file 41380_2018_255_MOESM1_ESM.docx]

**Supplementary Figures**

**Supplementary Figure 1. Schematic overview of the analyses performed to validate *in vivo* in mice the driver role of *TYROBP* in sporadic late onset Alzheimer’s disease (LOAD). (a)** We previously performed a multiscale gene network analysis of human brain specimens from visual cortex (VC), dorsolateral PFC, and cerebellum (CB) in 376 AD patients and 173 non-demented controls. We identified *TYROBP* as a key driver gene in the pathogenesis of sporadic LOAD^5^. **(b)** We generated WT or *APP/PSEN1* mice that are either WT, heterozygous- or homozygous-null for *Tyrobp*. At 8-months-old, we performed RNA sequencing on the prefontral cortices of the six groups of mice. The mouse molecular signatures were projected onto the human LOAD molecular signature to evaluate the degree to which the *Tyrobp* mouse model predicted LOAD perturbation signatures. We performed molecular, behavioral and synaptic plasticity assays to evaluate the functional role of TYROBP in WT and amyloidogenic context.

**Supplementary Figure 2. Gene expression of *TYROBP* in LOAD.** *TYROBP* mRNA is upregulated in brains from patients with dementia in 4 brain regions profiled by RNA-seq in the MSBB AD cohort. BM10: frontopolar prefrontal cortex; BM22: superior temporal gyrus; BM36: parahippocampal gyrus; BM44: inferior frontal gyrus.

**Supplementary Figure 3. RNA sequencing. (a)** Number of differentially expressed genes in 4 and 8-month-old mice WT, deficient or KO for TYROBP in *APP/PSEN1* or WT background. **(b)** Heatmap of dysregulated canonical pathways in PFCs of *APP/PSEN1;Tyrobp^-/-^ vs.* *APP/PSEN1* mice at 4- and 8-month-old of age. Data presented as -log(FDR). **(c)** List of the 86 shared DEGs (FDR 0.05) between *APP/PSEN1 vs.* WT and *APP/PSEN1;Tyrobp*^-/-^ *vs. APP/PSEN1.* **(d)** Comparison of the shared and unique DEGs (FDR 0.05) in *APP/PSEN1, APP/PSEN1;Tyrobp*^+/-^ and *APP/PSEN1;Tyrobp*^-/-^ mice. (up: upregulated; down: downregulated genes)

**Supplementary Figure 4. C1q mRNA and protein levels are reduced in *APP/PSEN1* mice that are constitutively deficient in TYROBP*.* (a)** qPCR analysis of C1q mRNA expression in frontal cortices from 8-month-old male and female *WT* (n=9)*,* *APP/PSEN1* (n=9)*, APP/PSEN1;Tyrobp^+/-^* (n=8) and *APP/PSEN1;Tyrobp^-/-^* (n=9) mice. One-way ANOVA corrected for multiple comparisons (Tukey) was used for statistical comparisons. ^$^p<0.05; ^$$^p<0.01 *vs*. WT. ****p < 0.0001 *vs. APP/PSEN1*. Data presented as mean±SEM. **(b)** Western blot analysis of C1q and GAPDH proteins in brain protein homogenates from 8-month-old male and female *APP/PSEN1, APP/PSEN1;Tyrobp^+/-^* and *APP/PSEN1;Tyrobp^-/-^* mice. Representative immunoreactive bands for C1q and GAPDH proteins. **(c)** Integrated density of immunoreactive western blot bands were measured for C1q and normalized on GAPDH (n=8/group). At least 2 independent western blot analyses were performed. One-way ANOVA corrected for multiple comparisons (Tukey) was used for statistical comparisons, *p<0.05; ****p<0.0001. Data presented as mean ± SEM. **(d)** Representative immunohistological staining of C1q (frontal cortex) in *APP/PSEN1* and *APP/PSEN1;Tyrobp^-/-^* mice.

**Supplementary Figure 5.** Genes upregulated in *APP/PSEN1* mice are enriched in the subnetwork neighboring TYROBP (9.9-fold, FET P value 4.3×10^-71^). Red color denotes upregulated genes while yellow denotes genes without significant change in expression.

**Supplementary Figure 6. Absence of TYROBP increased the levels of Aß in TBS fraction but decreased the levels of Aß in Triton-X fraction in 8-month-old *APP/PSEN1* mice. (a)** Schematic overview of the mouse genotypes and experiment. Aß40, Aß42, and oligomer levels were measured in hemibrains of female and male *APP/PSEN1* and *APP/PSEN1;Tyrobp^-/-^* mice. Hemibrains were processed via differential detergent solubilization to produce fractions of TBS soluble, Triton-X soluble and formic acid soluble Aβ. Levels of Aß40 **(b,e,h)** and Aß42 **(c,f,i)** were determined from each fraction via ELISA. The Aβ42/40 ratio was calculated for each fraction **(d,g,j)**. **(k,l,m)** Oligomeric Aβ was assessed from the TBS-soluble fraction via dot blot analyses using NU-4 **(k)**, A11 **(l)** and OC **(m)** antibodies. Mann-Whitney test. n=4-5 per group per sex. *p<0.05; **p<0.01; ***p<0.001. ns: not significant. Data presented as mean ± SEM.

**Supplementary Files**

**Supplementary Table 1:** Differential gene expression (DE) results

**Supplementary Table 2:** Gene set enrichment analysis results

**Supplementary Table 3:** Intersection analysis of *APP/PSEN1* mouse signature with multiple AD gene expression signatures previously identified in human brains. Intersection analysis results were obtained from the Fisher’s exact test (FET).

**Supplementary Table 4:** Intersection of mouse gene signatures on to co-expression network modules identified across multiple human AD datasets.

**Supplementary Methods**
