# Supplementary figures and images for: Integrative approach to sporadic Alzheimer’s disease: deficiency of TYROBP in cerebral Aβ amyloidosis mouse normalizes clinical phenotype and complement subnetwork molecular pathology without reducing Aβ burden
Source: Mol Psychiatry. 2018 Oct 3;24(3):431–46. doi: 10.1038/s41380-018-0255-6 (PMC6494440; doi:10.1038/s41380-018-0255-6)

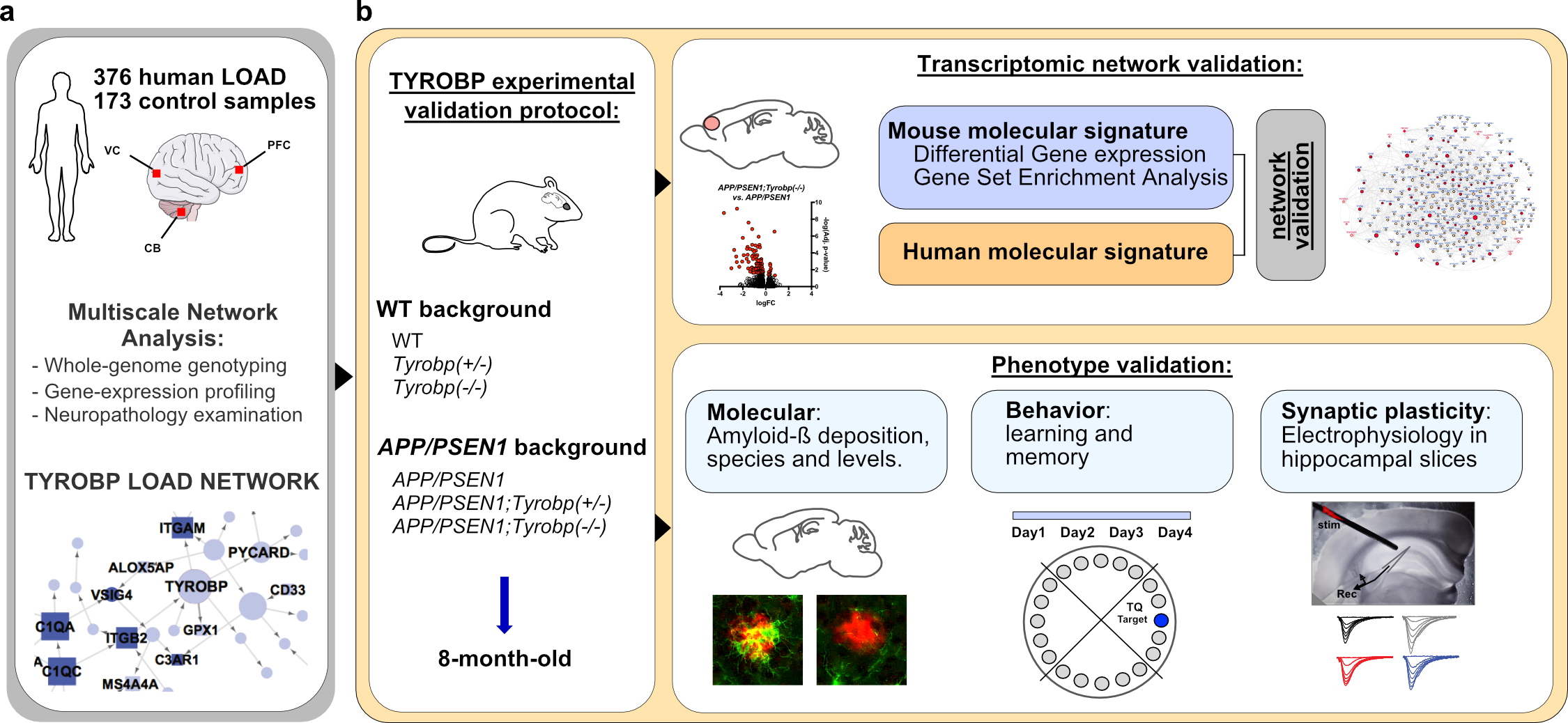

Supplement: Supplementary file 2 — Supplementary Figure 1 [file 41380_2018_255_MOESM2_ESM.tif]

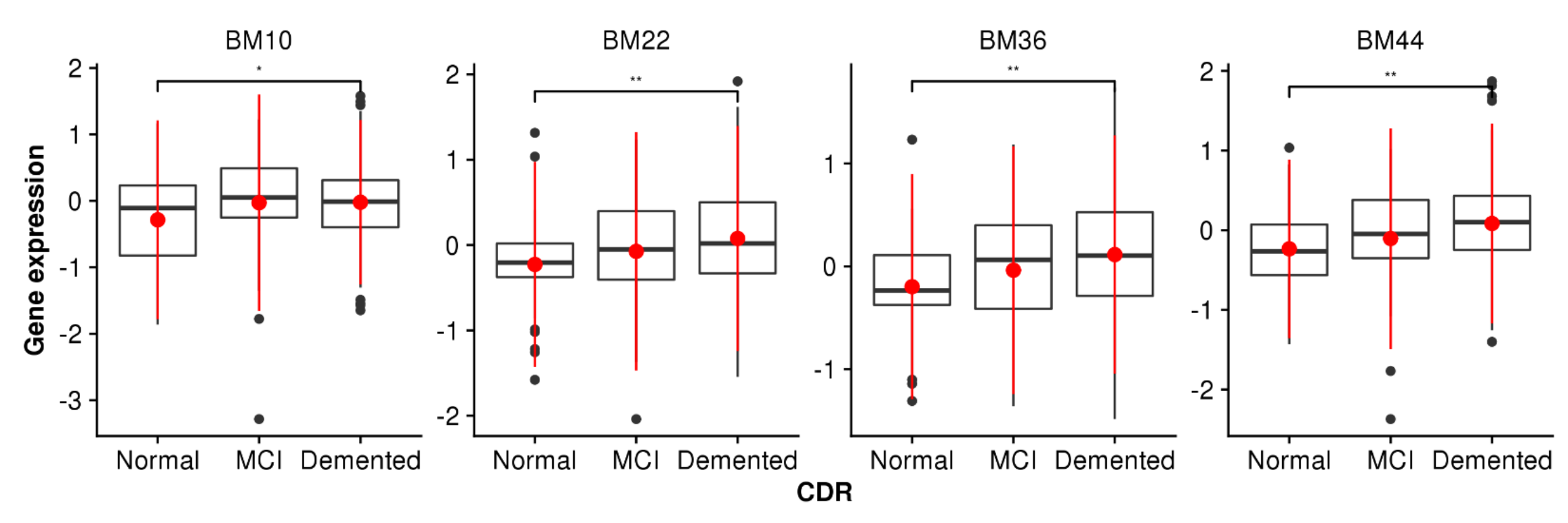

Supplement: Supplementary file 3 — Supplementary Figure 2 [file 41380_2018_255_MOESM3_ESM.tif]

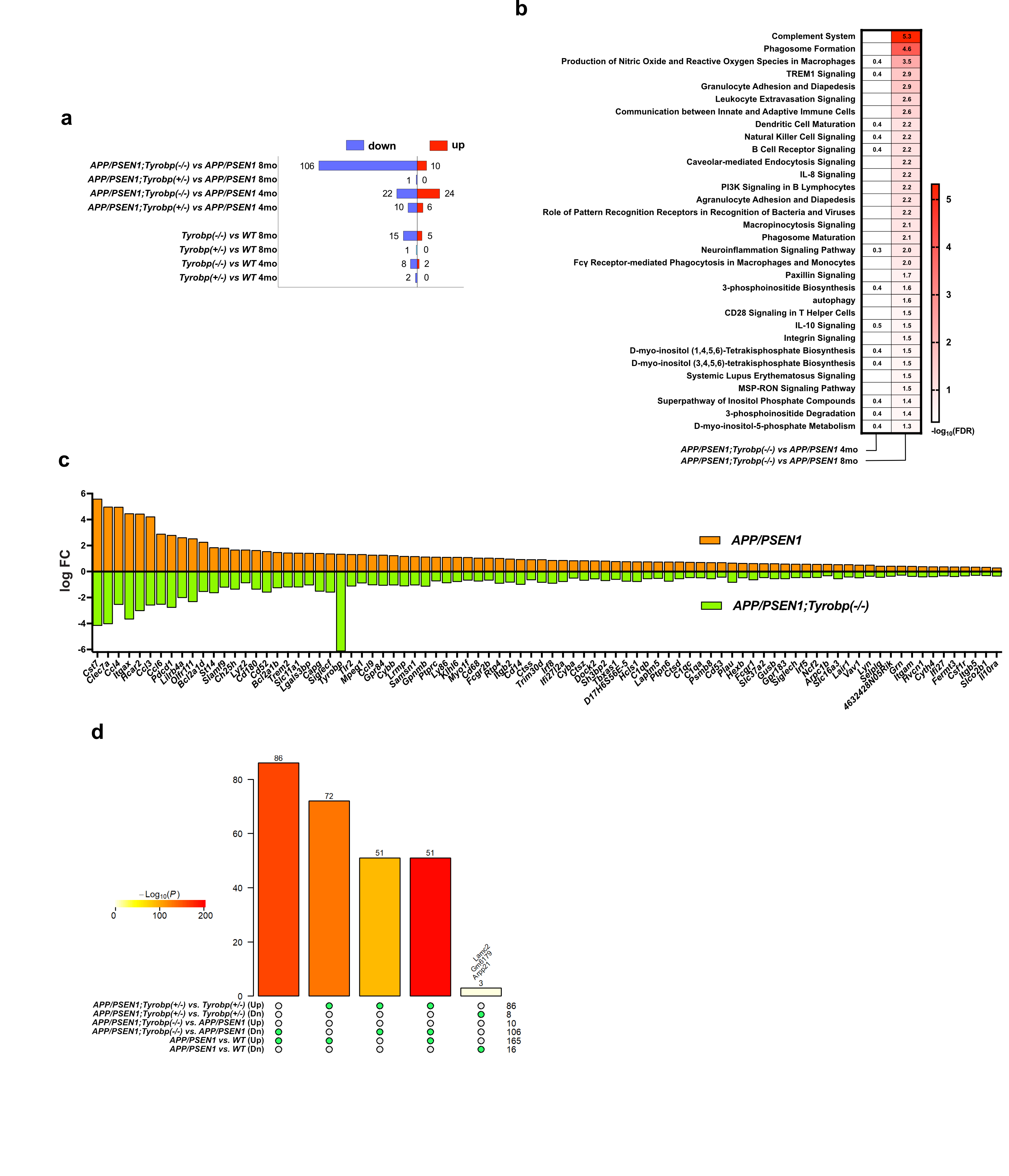

Supplement: Supplementary file 4 — Supplementary Figure 3 [file 41380_2018_255_MOESM4_ESM.tif]

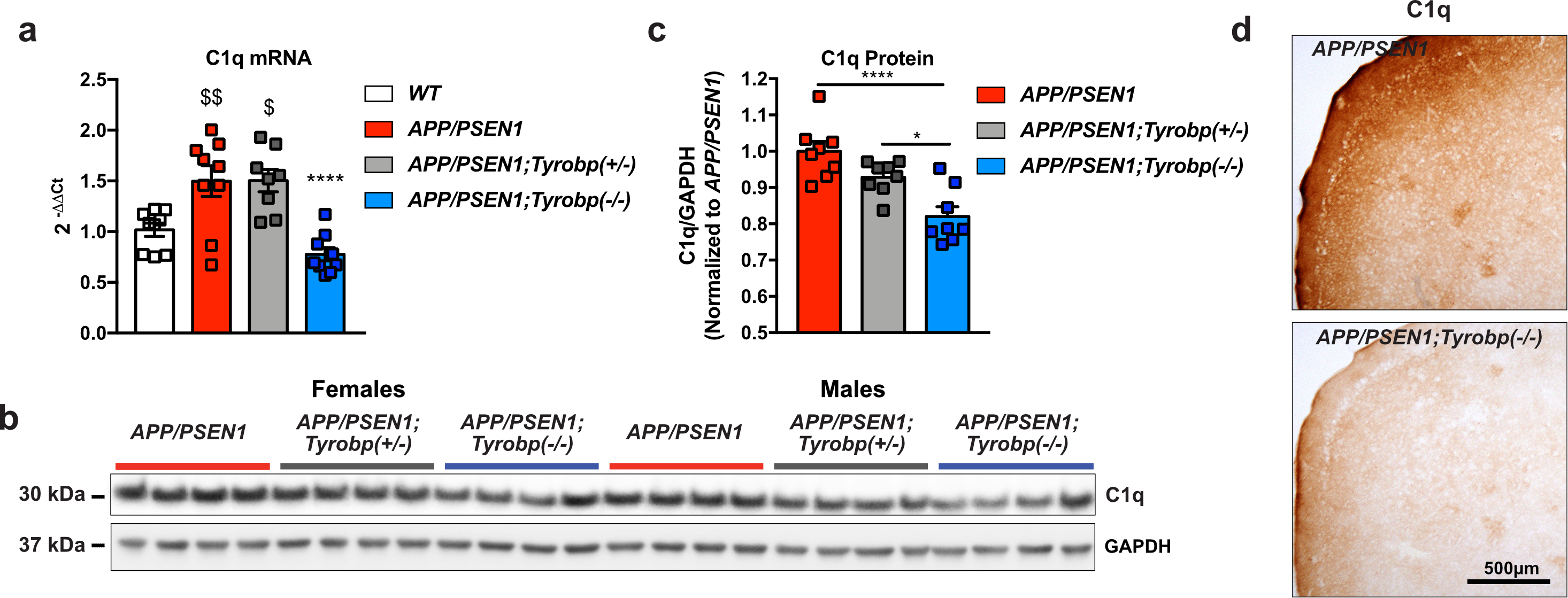

Supplement: Supplementary file 5 — Supplementary Figure 4 [file 41380_2018_255_MOESM5_ESM.tif]

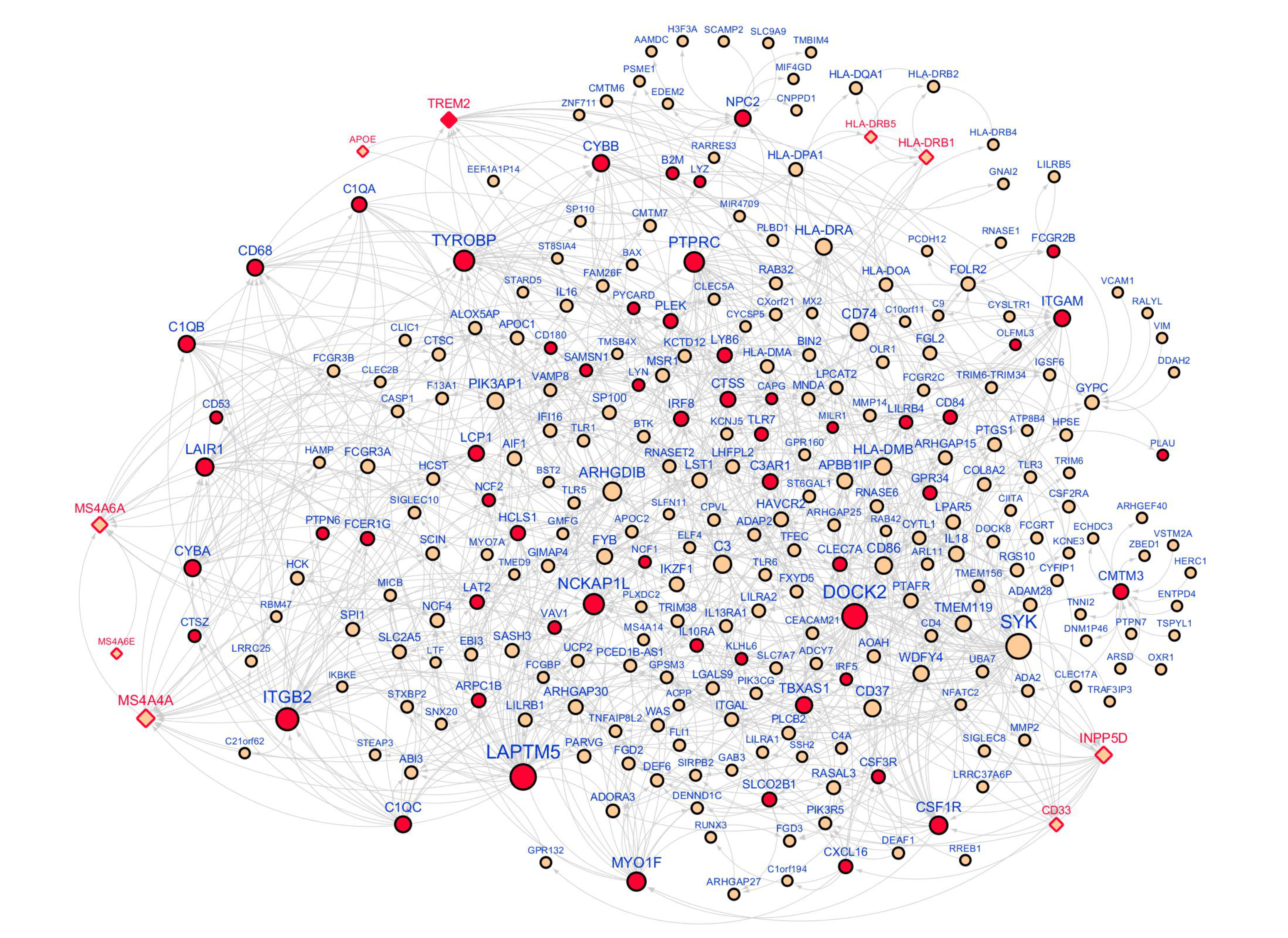

Supplement: Supplementary file 6 — Supplementary Figure 5 [file 41380_2018_255_MOESM6_ESM.tif]

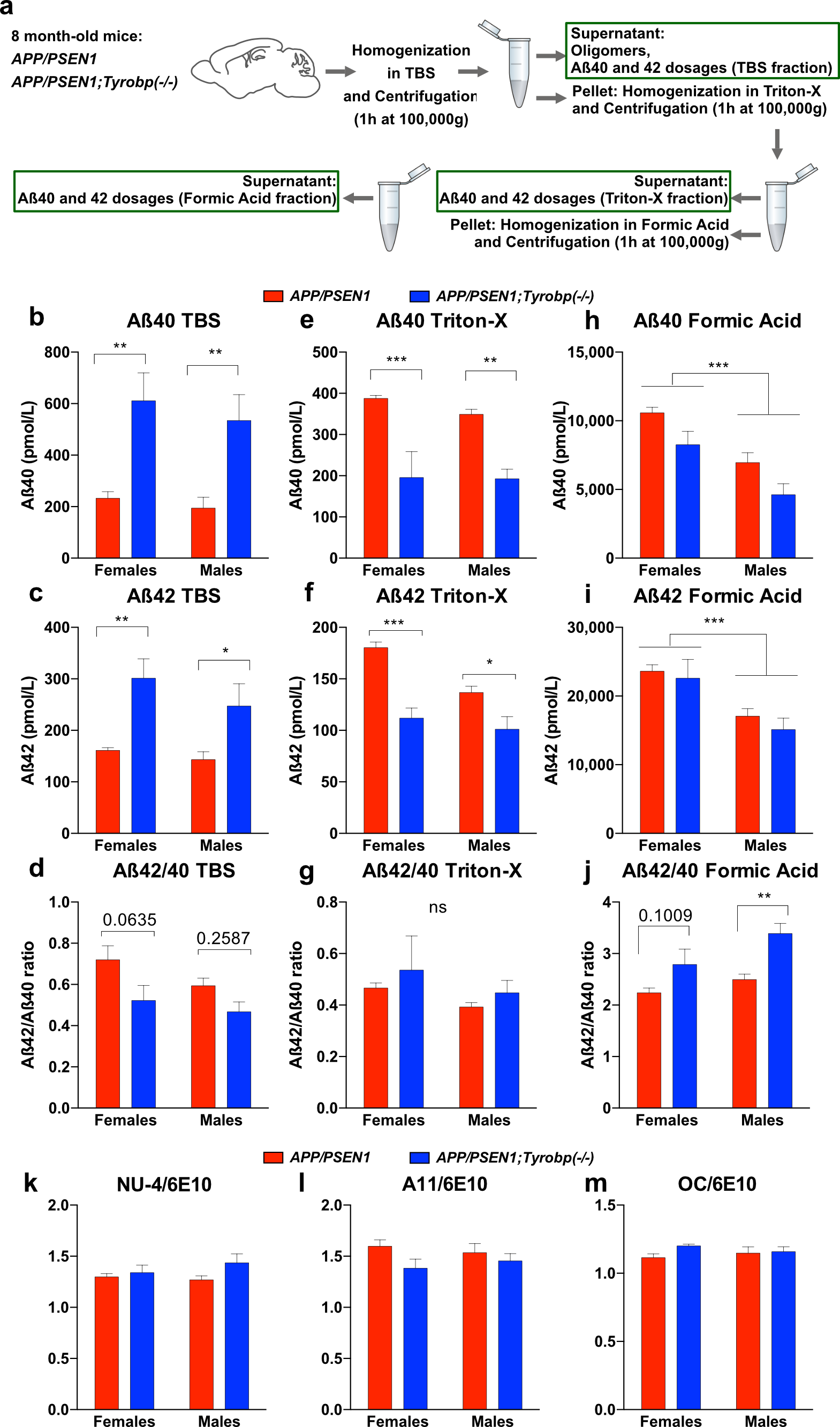

Supplement: Supplementary file 7 — Supplementary Figure 6 [file 41380_2018_255_MOESM7_ESM.tif]
