# Supplementary material for: Integrative approach to sporadic Alzheimer’s disease: deficiency of TYROBP in cerebral Aβ amyloidosis mouse normalizes clinical phenotype and complement subnetwork molecular pathology without reducing Aβ burden
Source: Mol Psychiatry. 2018 Oct 3;24(3):431–46. doi: 10.1038/s41380-018-0255-6 (PMC6494440; doi:10.1038/s41380-018-0255-6)
Supplement: Supplementary file 10 — Supplementary Table 3 [file 41380_2018_255_MOESM10_ESM.docx]

**Suppl. Table 3: Intersection analysis of *APP/PSEN1* mouse signature with multiple AD gene expression signatures previously identified in human brains. Intersection analysis results were obtained from the Fisher’s exact test (FET).**

| **HumanDEGs** | **MouseDEGs** | **Overlap#** | **MouseDEGs#** | **HumanDEGs#** | **FE** | **Pvalue** | **P.adj** |
| --- | --- | --- | --- | --- | --- | --- | --- |
| Miller_2013.up.in.HippCA3 | *APP/PSEN1 vs* WT (Up) | 16 | 151 | 354 | 4.1 | 1.69E-06 | 0.000614 |
| Miller_2013.up.in.HippCA1 | *APP/PSEN1 vs* WT (Up) | 19 | 151 | 524 | 3.3 | 4.53E-06 | 0.000811 |
| Miller_2013.up.in.HippCA3 | *APP/PSEN1;Tyrobp(+/-) vs Tyrobp (+/-)* (Up) | 11 | 81 | 354 | 5.3 | 6.68E-06 | 0.000811 |
| Miller_2013.up.in.HippCA1 | *APP/PSEN1;Tyrobp(+/-) vs Tyrobp (+/-)* (Up) | 13 | 81 | 524 | 4.2 | 1.05E-05 | 0.000957 |
| Zhang_2013.Pos.cor.w.Braak.in.PFC | *APP/PSEN1 vs* WT (Up) | 27 | 151 | 1040 | 2.4 | 2.14E-05 | 0.001558 |
| Zhang_2013.Pos.cor.w.Braak.in.PFC | *APP/PSEN1;Tyrobp(+/-) vs Tyrobp(+/-)* (Up) | 17 | 81 | 1040 | 2.8 | 9.31E-05 | 0.005647 |
| Miller_2013.up.in.HippCA3 | *APP/PSEN1;Tyrobp(-/-) vs* WT(Up) | 5 | 24 | 354 | 8.1 | 0.000309 | 0.016071 |
| Zhang_2013.Pos.cor.w.Braak.in.CB | *APP/PSEN1;Tyrobp(+/-) vs Tyrobp(+/-)* (Dn) | 3 | 6 | 398 | 17.3 | 0.000448 | 0.020393 |
| Miller_2013.up.in.HippCA3 | *APP/PSEN1;Tyrobp(-/-) vs APP/PSEN1* (Dn) | 9 | 101 | 354 | 3.5 | 0.001151 | 0.046567 |
