# Supplementary material for: Integrative approach to sporadic Alzheimer’s disease: deficiency of TYROBP in cerebral Aβ amyloidosis mouse normalizes clinical phenotype and complement subnetwork molecular pathology without reducing Aβ burden
Source: Mol Psychiatry. 2018 Oct 3;24(3):431–46. doi: 10.1038/s41380-018-0255-6 (PMC6494440; doi:10.1038/s41380-018-0255-6)
Supplement: Supplementary file 11 — Supplementary Table 4 [file 41380_2018_255_MOESM11_ESM.docx]

**Supplementary Table 4: Intersection of mouse gene signatures on to co-expression network modules identified across multiple human AD datasets.**

| **Dataset** | **Module** | **Annotation** | **Rank** | **DEGs** | **#Overlap** | **#DEGs** | **#Module** | **FE** | **Pvalue** | **P.adj** |
| --- | --- | --- | --- | --- | --- | --- | --- | --- | --- | --- |
| HBTRC | lightcyan | defense response | 25 | *APP/PSEN1 vs* WT (Up) | 76 | 151 | 420 | 22.8 | 6.25E-86 | 9.80E-83 |
| HBTRC | gold | response to biotic stimulus | 45 | *APP/PSEN1 vs* WT (Up) | 64 | 151 | 315 | 25.6 | 1.36E-74 | 1.07E-71 |
| HBTRC | yellow | response to biotic stimulus | 1 | *APP/PSEN1 vs* WT (Up) | 79 | 151 | 839 | 11.8 | 2.11E-66 | 1.10E-63 |
| HBTRC | lightcyan | defense response | 25 | *APP/PSEN1;Tyrobp(-/-) vs APP/PSEN1* (Dn) | 53 | 100 | 420 | 24 | 2.22E-61 | 8.70E-59 |
| HBTRC | gold | response to biotic stimulus | 45 | *APP/PSEN1;Tyrobp(-/-) vs APP/PSEN1* (Dn) | 47 | 100 | 315 | 28.3 | 2.45E-57 | 7.68E-55 |
| HBTRC | yellow | response to biotic stimulus | 1 | *APP/PSEN1;Tyrobp(-/-) vs APP/PSEN1* (Dn) | 51 | 100 | 839 | 11.5 | 2.34E-42 | 4.59E-40 |
| HBTRC | cyan | response to external biotic stimulus | 7 | *APP/PSEN1 vs* WT (Up) | 15 | 151 | 479 | 3.9 | 6.67E-06 | 0.001047 |
| ROSMAP | lightcyan | inflammatory response | 8 | *APP/PSEN1 vs* WT (Up) | 59 | 151 | 281 | 26.4 | 2.91E-69 | 1.46E-66 |
| ROSMAP | lightcyan | inflammatory response | 8 | *APP/PSEN1;Tyrobp(-/-) vs APP/PSEN1* (Dn) | 45 | 100 | 281 | 30.4 | 3.61E-56 | 9.09E-54 |
| ROSMAP | forestgreen | response to virus | 26 | *APP/PSEN1 vs* WT (Up) | 10 | 151 | 85 | 14.8 | 1.41E-09 | 1.77E-07 |
| ROSMAP | salmon | inflammatory response | 7 | *APP/PSEN1 vs* WT (Up) | 15 | 151 | 333 | 5.7 | 6.98E-08 | 7.03E-06 |
| MSBB.BM22 | green | inflammatory response | 3 | *APP/PSEN1 vs* WT (Up) | 87 | 151 | 789 | 13.9 | 1.68E-80 | 4.81E-77 |
| MSBB.BM44 | brown | inflammatory response | 4 | *APP/PSEN1 vs* WT (Up) | 92 | 151 | 1033 | 11.2 | 2.27E-77 | 3.24E-74 |
| MSBB.BM10 | lightyellow | inflammatory response | 15 | *APP/PSEN1 vs* WT (Up) | 59 | 151 | 238 | 31.2 | 5.41E-74 | 5.15E-71 |
| MSBB.BM22 | green | inflammatory response | 3 | *APP/PSEN1;Tyrobp(-/-) vs APP/PSEN1* (Dn) | 61 | 100 | 789 | 14.7 | 1.09E-58 | 7.81E-56 |
| MSBB.BM10 | lightyellow | inflammatory response | 15 | *APP/PSEN1;Tyrobp(-/-) vs APP/PSEN1* (Dn) | 44 | 100 | 238 | 35.1 | 8.73E-58 | 4.99E-55 |
| MSBB.BM36 | green | inflammatory response | 3 | *APP/PSEN1 vs* WT (Up) | 64 | 151 | 644 | 12.5 | 8.51E-54 | 4.05E-51 |
| MSBB.BM44 | brown | inflammatory response | 4 | *APP/PSEN1;Tyrobp(-/-) vs APP/PSEN1* (Dn) | 60 | 100 | 1033 | 11 | 4.48E-50 | 1.83E-47 |
| MSBB.BM36 | green | inflammatory response | 3 | *APP/PSEN1;Tyrobp(-/-) vs APP/PSEN1* (Dn) | 44 | 100 | 644 | 13 | 4.28E-38 | 1.22E-35 |
| MSBB.BM10 | tan | inflammatory response | 24 | *APP/PSEN1 vs* WT (Up) | 25 | 151 | 435 | 7.2 | 8.31E-15 | 1.83E-12 |
| MSBB.BM10 | tan | inflammatory response | 24 | *APP/PSEN1;Tyrobp(-/-) vs APP/PSEN1* (Dn) | 13 | 100 | 435 | 5.7 | 4.66E-07 | 9.50E-05 |
