# Supplementary material for: Integrative approach to sporadic Alzheimer’s disease: deficiency of TYROBP in cerebral Aβ amyloidosis mouse normalizes clinical phenotype and complement subnetwork molecular pathology without reducing Aβ burden
Source: Mol Psychiatry. 2018 Oct 3;24(3):431–46. doi: 10.1038/s41380-018-0255-6 (PMC6494440; doi:10.1038/s41380-018-0255-6)
Supplement: Supplementary file 12 — Supplementary Methods [file 41380_2018_255_MOESM12_ESM.docx]

**Supplementary Methods**

**Human sporadic LOAD postmortem brain gene expression network analysis**

We obtained human postmortem brain gene expression data from two independent LOAD cohorts (MSBB^5,8^ and ROSMAP^44,45^) from the AMP-AD Knowledge Portal (https://www.synapse.org/#!Synapse:syn2580853). There are 4 brain regions profiled in the MSBB cohort and 1 brain region in the ROSMAP cohort. In each cohort, genes expressed in at least 10% of the samples were selected and then the normalized data were corrected for confounding factors including batch, PMI, sex, and RIN score, by using a linear regression. After data preprocessing, gene co-expression network was first constructed for each brain region by using R package WINA^5^, which implements a computationally optimized procedure for weighted gene co-expression network analysis^8^. In WINA analysis, we used power *β* = 6 with other parameters set by default. Modules (i.e., clusters of genes showing highly correlated expression profiles across samples) were named by arbitrary colors and annotated by the most highly enriched gene ontology/canonical pathway term. The modules were rank-sorted in relation to AD pathology by multiple features following an ensemble sorting metric^5^, including module-trait correlations and enrichment for AD related disease gene signatures including differentially expressed genes (DEGs) and trait-correlated genes regarding all available neuropathological/clinical traits including Braak staging, cognitive score, CERAD neuropathological category, and plaque density.

A Bayesian causal network was constructed separately for each brain region from genome-wide gene expression data. To assist with the inference of causal relations between genes, genotypes from whole-exome sequencing (WES) were used to compute the prior probability of causal links for the MSBB data. For the ROSMAP data, genome-wide SNP genotype and DNA methylation were also used to infer the prior probability of causal links. Briefly, we first computed expression quantitative trait loci (eQTLs) based on associations between gene expression and SNP or WES genotype data by using R package MatrixEQTL and then employed a formal statistical causal inference test (CIT)^61^ to infer the causal probability between gene pairs associated with the same eQTL. With a similar strategy, we also computed methylation QTLs (mQTLs) and inferred causal probability of gene pairs mediated by DNA methylation for the ROSMAP data. The causal relationships inferred from eQTLs and mQTLs were combined with known transcription factor (TF)-target relationships obtained from ENCODE, and together they were subsequently used as priors for building a causal network through a Monte Carlo Markov Chain (MCMC) simulation based procedure^61^. After construction of individual networks, Bayesian networks previously constructed from the HBTRC gene expression data^61^ were merged by combining directed links with the present Bayesian networks to create a union Bayesian network.

**Immunohistochemistry.**

30 µm thick sagittal free-floating sections were pre-treated with 70% formic acid for 15min. Sections were blocked for 1h (10% goat serum, PBS, 0.3% Triton-X) and incubated overnight at 4°C with anti-Iba1 (1:500; cat# 019-19741, Wako, Richmond, VA, USA), 6E10 (1:1000; cat# 9320-500, Covance, Princeton, NJ, USA), or C1q (1:1000, cat# ab182451, Abcam, Cambridge, MA, USA) antibodies (1% goat serum, PBS, 0.1% Tween-20 =PBS-T). Sections probed with Iba1 and 6E10 antibodies were washed (PBS-T) and incubated for 1h with fluorescent conjugated secondary antibodies (1% goat serum, PBS-T) (anti-rabbit Alexa 488 (1:400, cat#A-11008, Thermo Fisher Scientific, Grand Island, NY, USA) for Iba1 and anti-mouse Alexa 594 (1:400, cat#A-11005, Thermo Fisher Scientific) for 6E10). Sections probed with C1q were washed (PBS-T), incubated for 1h with biotinylated anti-mouse antibody (1:1000, BA-9200, Vector laboratories, Burlingame, CA, USA) (1% goat serum, PBS-T) and developed with Vectastain ABC Kit (PK-4000, Vector Laboratories).

**Western Blot.**

30µg of protein lysates prepared from hemibrains homogenized in RIPA buffer (150mM NaCl, 0.1% Triton-X, 0.5% sodium deoxycholate, 0.1% SDS, 50mM Tris-HCL pH=8.0) were loaded in Criterion XT 4-20% Bis-Tris gels and transferred onto activated/pre-wetted PVDF membrane (0.45μm; Millipore, Billerica, MA, USA). Membranes were blocked for 1h (5% non-fat milk, TBS-T) and probed overnight at 4°C with anti-C1q antibody (1:1000, cat# ab182451, Abcam) in blocking buffer. Membranes were washed (TBS-T), incubated with anti-rabbit HRP-conjugated secondary antibodies (1:2000, cat#PI-1000, Vector laboratories) for 1h in blocking buffer, washed again (TBS-T) and developed with ECL Western blotting substrate (Pierce, Rockford, IL, USA) using the Fujifilm LAS-3000 developer (Stamford, CT, USA). Normalization was achieved using anti-GAPDH antibody. Thus, membranes were stripped (Restore PLUS Western Blot Stripping Buffer, Thermo Scientific), washed, blocked 1h, probed 1h with anti-GAPDH antibody (1:5000, cat# sc32233, Cruz Biotechnology, Dallas, TX, USA) in blocking buffer, and incubated with anti-mouse HRP-conjugated secondary antibodies (1:2000, cat#PI-2000, Vector laboratories) for 1h in blocking buffer. Membranes were developed as described above. Integrated density of immunoreactive bands were measured using MultiGauge Software (FujiFilm).

**Serial detergent fractionation with ultracentrifugation for Aß assays and oligomer epitope characterization.**

Hemibrains were processed via serial detergent fractionation with ultracentrifugation to produce TBS-soluble, Triton-X-soluble and formic-acid-soluble Aβ fractions. Hemibrains were homogenized in ice-cold TBS (pH=7.6) (200mg tissue/1mL TBS) containing protease/phosphatase inhibitor cocktail (#78443, Pierce). Homogenates were ultracentrifuged at 100,000xg for 1h at 4°C and the supernatants were removed and stored at −80°C until analysis (TBS fraction). The TBS-insoluble pellets were then homogenized in Triton-X solution (TBS 1% (v/v) Triton-X-100) containing protease/phosphatase inhibitor cocktail and ultracentrifuged as above. The supernatants were removed and stored at -80°C until analysis (Triton-X fraction). The Triton-X-insoluble pellets were then homogenized in 70% formic acid, ultracentrifuged as above, neutralized by 1:20 dilution into 1M Tris (pH=11.0), and stored at -80°C (Formic acid fraction) (See **Suppl. Figure 5a**). All homogenization and sample collection steps were performed on ice, and samples were aliquoted to avoid denaturing of protein by repeated freeze–thaw cycles.

**Aß assays and oligomer epitope characterization.**

For analysis of native oligomeric Aβ protein structure, 2μl protein samples from the TBS-soluble fraction were spotted onto activated PVDF membrane (0.22μm; Millipore). Membranes were blocked for 1h (5% non-fat milk, TBS-T) and incubated overnight at 4°C in blocking buffer with either rabbit pAb A11 (anti-prefibrillar oligomers; 0.5μg/ml; gift from Charles Glabe, University of California Irvine), rabbit pAb OC (anti-fibrillar oligomers and fibrils; 0.25μg/ml; gift from Charles Glabe) or mouse mAb NU-4 (anti-oligomers; 1μg/ml; gift from William Klein, Northwestern University)^28,39,40^. Generation, purification, and characterization of A11, OC and NU-4 have been described previously^39,40^. Membranes were incubated with appropriate anti-mouse or -rabbit HRP-conjugated secondary antibody (1:20,000; Vector laboratories) for 1h in blocking buffer and developed as described in the western section. Normalization to total APP/Aβ signal was achieved by detection of human APP transgene metabolites with the anti-Aß antibody 6E10 (1:1000; Covance). Thus, membranes were stripped in low pH stripping buffer (25mM Glycine HCl, pH=2.0 and 1% w/v SDS), blocked 1h and probed overnight at 4°C in blocking buffer with mouse 6E10 antibody. Membranes were then incubated in anti-mouse HRP-conjugated secondary antibody for 1h in blocking buffer and developed as described above.

**Antibodies used:**

anti-Iba1: Wako, cat#019-19741, lot#WDK2121; validated for IHC in human, mouse and rat.

anti-6E10: Covance, cat#9320-500, lot#D14FF01323; validated for Elisa, WB, IHC, IP and reactive to amino acid residue 1-16 of beta amyloid.

anti-C1q: Abcam, cat#ab182451, lot#GR290355-11, Clone 4.8; validated for IHC in mouse.

anti-GAPDH: Santa Cruz, cat#sc32233, lot#H2114, clone 6C5; validated for WB, IP, IHC in mouse, rat, human, rabbit and Xenopus laevis.

anti-NU-4: anti-oligomers; gift from William Klein, Northwestern University; characterized by Lambert et al. and others for WB, IHC and dot blot.

anti-OC: anti-fibrils and fibrillar oligomers; gift from Charles Glabe, University of California Irvine; characterized by Tomic et al, 2009 and others for WB and dot blot assay.

anti-A11: anti-prefibrillar oligomers; gift from Charles Glabe, University of California Irvine; characterized by Tomic et al, 2009 and others for WB and dot blot assay.

anti-rabbit Alexa 488: Thermo Fisher Scientific, Cat#A-11008, lot#1885240.

anti-mouse Alexa 594: Thermo Fisher Scientific, Cat#A-11005, lot#1310420.

biotinylated anti-mouse antibody: Vector laboratories, Cat#BA-9200, lot#W0206.

anti-rabbit HRP-conjugated secondary antibodies: Vector laboratories, Cat#PI-1000, lot#ZC0304.

anti-mouse HRP-conjugated secondary antibodies: Vector laboratories, Cat#PI-2000, lot#ZC1212.
